# The NAC transcription factor MdNAC4 positively regulates nitrogen deficiency-induced leaf senescence by enhancing ABA biosynthesis in apple

**DOI:** 10.1186/s43897-023-00053-4

**Published:** 2023-03-10

**Authors:** Binbin Wen, Xuehui Zhao, Xingyao Gong, Wenzhe Zhao, Mingyue Sun, Xiude Chen, Dongmei Li, Ling Li, Wei Xiao

**Affiliations:** 1 State Key Laboratory of Crop Biology, College of Horticulture Science and Engineering, Shandong Agricultural University, Taian, 271018 Shandong China; 2 College of Seed and Facility Agricultural Engineering, Weifang University, Weifang, 261061 Shandong China

**Keywords:** Apple, MdNAC4, MdPYL4, N deficiency, ABA, Leaf senescence

## Abstract

Although it is well established that nitrogen (N) deficiency induces leaf senescence, the molecular mechanism of N deficiency-induced leaf senescence remains largely unknown. Here, we show that an abscisic acid (ABA)-responsive NAC transcription factor (TF) is involved in N deficiency-induced leaf senescence. The overexpression of *MdNAC4* led to increased ABA levels in apple calli by directly activating the transcription of the ABA biosynthesis gene *MdNCED2*. In addition, *MdNAC4* overexpression promoted N deficiency-induced leaf senescence. Further investigation showed that MdNAC4 directly bound the promoter of the senescence-associated gene (SAG) *MdSAG39* and upregulated its expression. Interestingly, the function of MdNAC4 in promoting N deficiency-induced leaf senescence was enhanced in the presence of ABA. Furthermore, we identified an interaction between the ABA receptor protein MdPYL4 and the MdNAC4 protein. Moreover, MdPYL4 showed a function similar to that of MdNAC4 in ABA-mediated N deficiency-induced leaf senescence. These findings suggest that ABA plays a central role in N deficiency-induced leaf senescence and that MdPYL4 interacts with MdNAC4 to enhance the response of the latter to N deficiency, thus promoting N deficiency-induced leaf senescence. In conclusion, our results provide new insight into how MdNAC4 regulates N deficiency-induced leaf senescence.

**Graphical Abstract:**

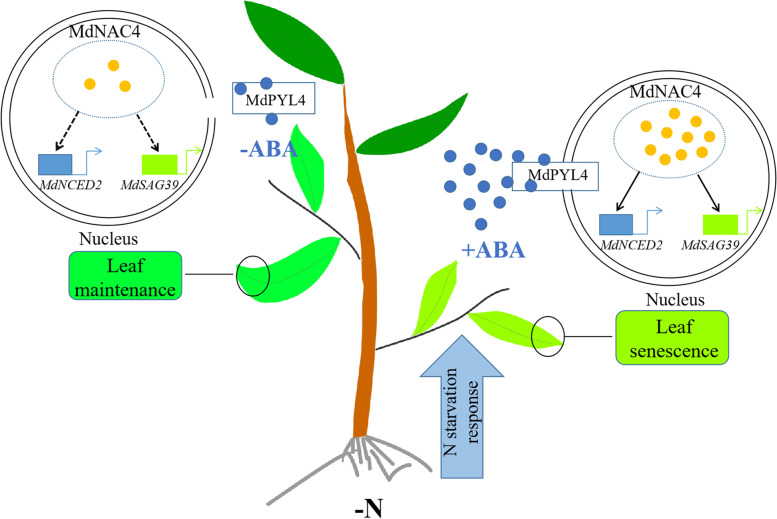

**Supplementary Information:**

The online version contains supplementary material available at 10.1186/s43897-023-00053-4.

## Core

N deficiency tends to induce rapid leaf senescence, and the apple NAC transcription factor MdNAC4 positively regulates N deficiency-induced leaf senescence through the ABA biosynthetic pathway. Further studies showed that the MdNAC4 protein interacts with the ABA receptor protein MdPYL4, which enhances the response of MdNAC4 to N deficiency and promotes N deficiency-induced apple leaf senescence.

## Gene & Accession Numbers

Gene sequence information was obtained from the apple (GDR https://www.rosaceae.org/) and tobacco (https://www.ncbi.nlm.nih.gov/) databases. The accession numbers of the genes used in this study are as follows: *MdNAC4* (MD17G1051600), *MdPYL4* (MD07G1227100), *MdNYC1* (MD02G1045900) *MdPAO* (MD11G1149200), *MdSGR1* (MD04G1070300), *MdSAG12* (MD16G1244200), *MdSAG29* (MD16G1125300), *MdSAG39* (MD00G1208000), *MdNCED1* (MD02G1309700), *MdNCED2* (MD10G1261000), *MdNCED6* (MD16G1235500), *MdCYP707A1* (MD16G1285900), *MdZEP* (MD02G1172400), *MdVNI2* (MD16G1125800), *MdSINA1* (MD12G1055100), *MdBFN1* (MD10G1079400), *NtNYC1* (XM_016652882.1), *NtPAO* (NM_001325995.1), *NtSGR1* (XM_016651072.1), *NtSAG12* (NM_001325416.1), *NtSAG29* (XM_016591710.1) and *NtSAG39* (XM_016605395.1).

## Introduction

Leaf senescence is a highly coordinated developmental process constituting the final stage of leaf development. During this period, the organelle structure of leaf cells is sequentially disorganized, and metabolism and gene expression change in an orderly manner (Woo et al. 2019). As leaves senesce, their carbon assimilation capacity and photosynthesis decline, while the degradation of chlorophyll, nucleic acids, proteins, and other macromolecules increases (Ischebeck et al. 2006; Lim et al. 2007). The increased catabolism activity that occurs results in the conversion of the cellular materials that accumulate during the leaf growth stage into exportable nutrients to meet the demand for nutrients in younger and developing tissues (Meng et al. 2016). Apple is a perennial deciduous fruit tree, whose leaf life has a great influence on the yield and quality of its fruit. Although leaf senescence promotes the reallocation and utilization of nutrients, it reduces the time period of photosynthesis and carbon assimilation, resulting in reductions in yield and quality (An et al. 2019; Woo et al. 2013).

ABA is a sesquiterpene phytohormone involved in the regulation of plant responses to abiotic and biotic stresses and various developmental processes. Previous studies have shown that ABA content increases with leaf senescence and that exogenous ABA promotes leaf senescence by inducing the expression of leaf SAGs (Oka et al. 2012; van der Graaff et al. 2006; Xie et al. 2021). NAC TFs are upregulated during leaf senescence and are involved in ABA-mediated leaf senescence (Gregersen and Holm 2007; Mao et al. 2017). In rice, the ABA-responsive NAC TFs NAC2 and NAC054 enhance the expression of SAGs by inducing the expression of ABA biosynthesis or signal transduction genes, thus promoting leaf senescence (Sakuraba et al. 2020; Mao et al. 2017). In addition, the *Arabidopsis* ABA-inducible TF NAP promotes leaf senescence by activating the expression of the phosphatase gene *SAG113* and the ABA biosynthesis gene *ABSCISIC ALDEHYDE OXIDASE3* (*AAO3*) (Yang et al. 2014; Zhang and Gan 2012), and the foxtail millet NAC transcription factor NAC1, which is induced by ABA and senescence signals, accelerates leaf senescence by promoting ABA biosynthesis (Ren et al. 2018). Overall, it is well established that ABA promotes leaf senescence, but the underlying mechanisms that regulate apple leaf senescence are poorly understood.

In recent years, studies on the ABA signaling pathway have revealed pyrabactin resistance/pyr1-like (PYR/PYL) proteins to be core components of the ABA signaling network mediating ABA-promoted leaf senescence (Miyakawa et al. 2013; Park et al. 2009; Zhao et al. 2016). The large-scale screening of transgenic plants overexpressing *PYL* family genes has shown that PYL9 promotes ABA-induced leaf senescence by inhibiting protein phosphatase 2Cs (PP2Cs) and activating SNF1-related kinases (SnRKs). Additionally, ABRE-binding factors (ABFs) 2/3/4 regulate the expression of SAGs and chlorophyll catabolism-related genes (CCGs) through the ABA signaling cascade pathway (PYLs–PP2C–SnRK2). Thus, PYLs play an important role in regulating ABA-induced leaf senescence and chlorophyll degradation.

N is a key macronutrient for plant growth and development and is an important component of N-containing compounds such as proteins, nucleotides, chlorophyll, hormones and various enzymes (Distelfeld et al. 2014; Marschner 1995); hence, N deficiency induces leaf senescence. Some studies have reported that N deficiency may interact with the soluble sugar level, amino acid content and antioxidant enzyme activity in a complex network to accelerate the process of leaf senescence (Agüera et al. 2010; Sultana et al. 2021; Srivalli and Khanna-Chopra 2009). In addition, plant internal systems or various hormones may sense low-N stress and transform it into molecular signals to induce the expression of SAGs. Under conditions of low-N stress, the content of strigolactone decreases, as does the expression level of NAC-S (Ito et al. 2016). This decrease in NAC-S levels leads to an increase in the expression of SAG CHLOROPHYLLASE 2 (*CLH2*), promoting leaf senescence (Yu et al. 2011; Sultana et al. 2021). *NITRATE TRANSPORTER 1.5* (*NRT1.5*), a xylem nitrate-loading transporter gene, is downregulated under low-N conditions and prevents leaf senescence by promoting the accumulation of foliar potassium (Meng et al. 2016). Moreover, the expression of *NRT1.5* is induced by the ethylene/jasmonic acid (ETH/JA) signaling pathway, and the ET/JA-NRT signaling module is considered to be an important module for plant adaptation to N stress (Lin et al. 2008; Zhang et al. 2014). Low-N conditions also interact with ABA to regulate plant senescence. The ABA level of cucumber (*Cucumis sativus*) plants grown under low N levels is significantly higher than that of cucumber plants grown under N-sufficient conditions (Oka et al. 2012). The ABA content of cotton was found to be significantly higher under low-N treatment than under normal-N treatment, and expression of the ABA biosynthesis genes *9-CIS-EPOXYCAROTENOID DIOXYGENASE 1* (*NCED1*) and *NCED6* was downregulated under the former conditions (Zhu et al. 2021). These results indicate that N deficiency interacts with multiple internal and external factors to regulate leaf senescence, although the underlying molecular mechanisms remain to be investigated.

In the present study, we identified and characterized an apple senescence-associated NAC TF, MdNAC4, and found that it is involved in the ABA signaling pathway. MdNAC4 activates the expression of the ABA biosynthesis-related gene *MdNCED2*, which leads to increased ABA levels. MdNAC4 also accelerates N deficiency-induced leaf senescence and directly activates the expression of the SAG *MdSAG39*. Moreover, we identified a protein that directly interacts with MdNAC4: MdPYL4. MdPYL4 has a similar function to MdNAC4 in ABA-mediated N deficiency-induced leaf senescence. In conclusion, this study elucidated a novel NAC transcription factor that positively regulates N deficiency-induced leaf senescence by enhancing ABA biosynthesis. The identification and characterization of MdNAC4 provides new insight into the molecular mechanism of N deficiency-induced leaf senescence.

## Results

### *MdNAC4* responds to ABA treatment

**Fig. 1:**
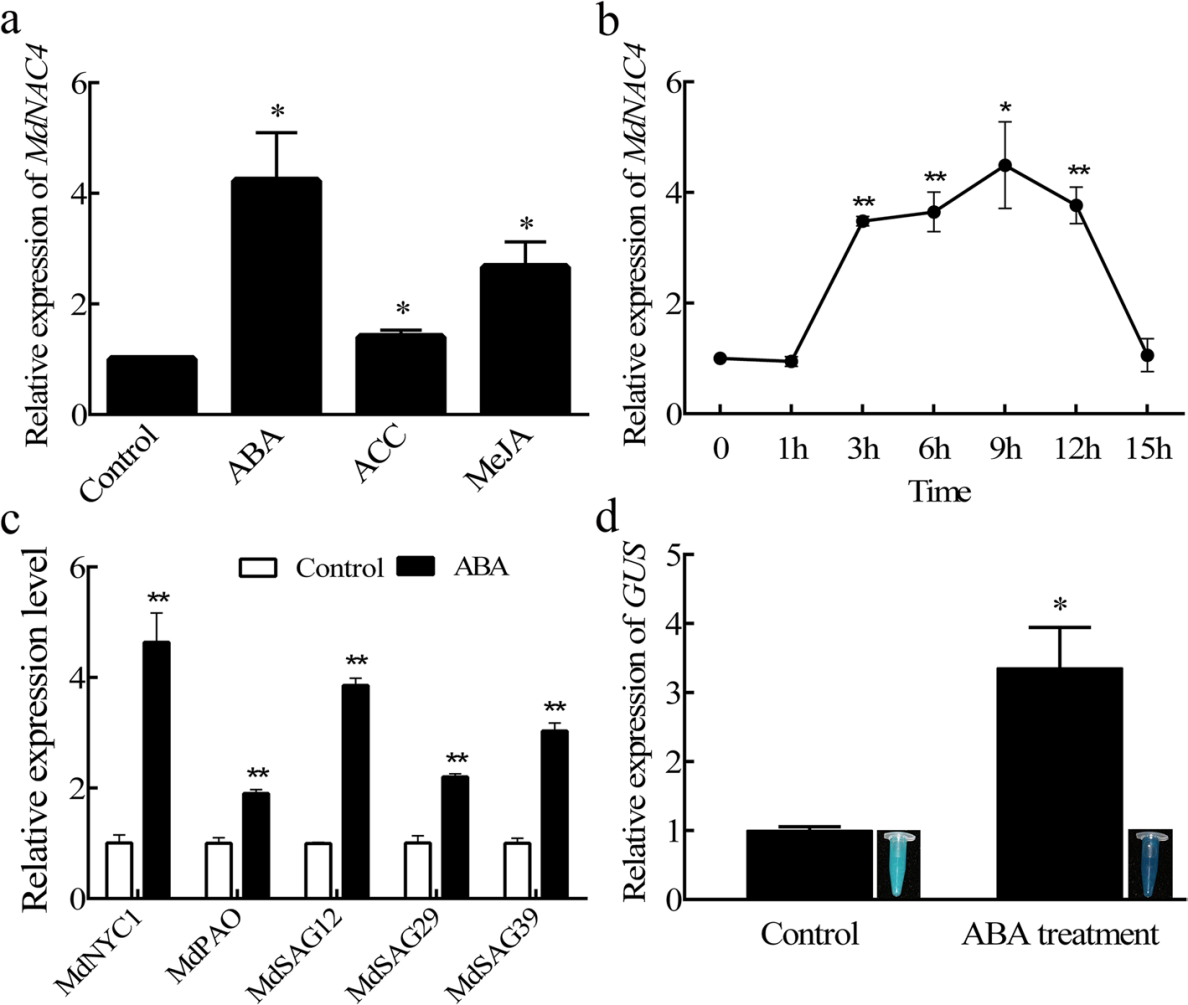
Response of *MdNAC4* to ABA treatment. **a** Expression levels of *MdNAC4* in apple seedling leaves treated with different phytohormones. Apple seedlings grown in the nutrient bowl were treated with 50 μM abscisic acid (ABA), 50 μM methyl jasmonate (MeJA), and 50 μM 1-aminocyclopropane-1-carboxylic acid (ACC) for 9 h by adding solutions containing ABA, ACC, and MEJA to the nutrient bowl. **b** Expression patterns of *MdNAC4* after 50 μM ABA treatment. Samples were taken at designated times (0, 1, 3, 6, 9, 12 and 15 h) for RNA extraction. **c** Expression levels of senescence-related genes (*MdNYC1*, *MdPAO*, *MdSAG12*, *MdSAG29* and *MdSAG39*) after 50 μM ABA treatment for 9 h. (d) GUS staining and qRT–PCR analysis of *MdNAC4* promoter transgenic apple calli. Control: no treatment; ABA treatment: 50 μM ABA treatment for 9 h. The expression level in untreated samples was set at 1. Error bars indicate the SDs of the three technical replicates and three biological replicates. Asterisks indicate significant differences between two independent samples according to t tests (*, *P* < 0.05 and **, *P* < 0.01)

Senescence is mainly regulated by developmental age, but the initiation and progression of this process are also regulated by a variety of hormones, including ABA, ETH and JA. Accordingly, *MdNAC4* could be induced by ABA, 1-aminocyclopropane-1-carboxylic acid (ACC), and methyl jasmonate (MeJA), but the induction of ABA was much more dramatic than that of ACC and MeJA (Fig. 1a). In addition, the expression of *MdNAC4* induced by 50 μm ABA seemed to reach a peak after 9-12 h of induction (Fig. 1b). To verify whether ABA induces the expression of other SAGs, the expression levels of SAGs (*MdNYC1*, *MdPAO*, *MdSAG12*, *MdSAG29*, and *MdSAG39*) were evaluated after ABA treatment, and the results revealed the induction of these SAGs (Fig. 1c). Finally, the *MdNAC4* promoter sequence was fused with the pCAMBIA1300-GUS expression vector, and transgenic apple calli were obtained by *Agrobacterium* infection. GUS staining and *GUS* gene expression analysis were performed on calli treated with ABA for 9 h. The results showed that GUS activity and expression levels were significantly increased after ABA treatment (Fig. 1d), which indicates that ABA induces the expression of *MdNAC4*.

### MdNAC4 increases ABA content by regulating the expression of ABA metabolic pathway genes

**Fig. 2:**
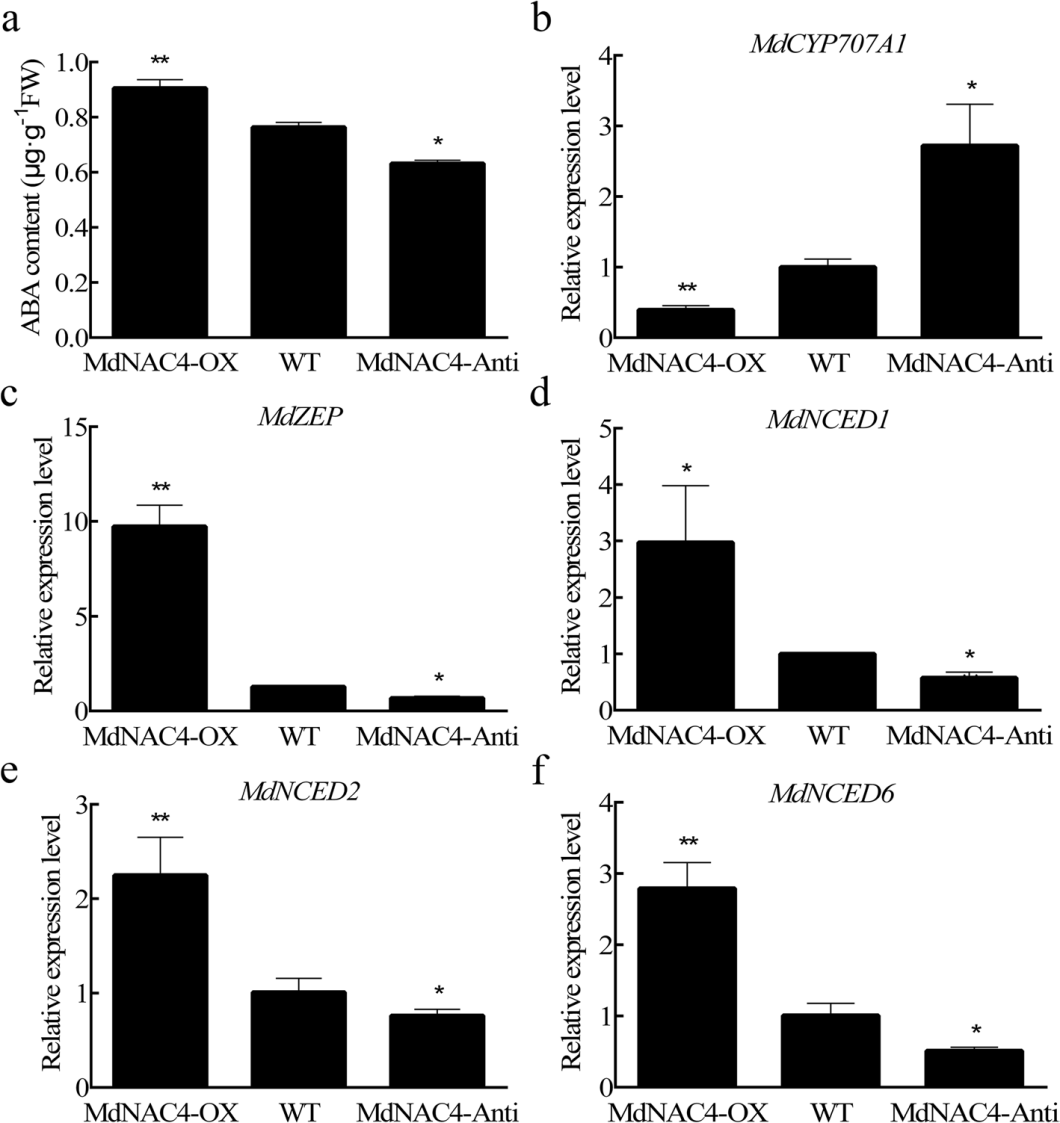
ABA content and expression levels of ABA metabolism-related genes in 2-week-old apple calli. **a** ABA content. **b-f** Expression levels of ABA metabolism-related genes. WT: wild-type; MdNAC4-OX: with MdNAC4 overexpression vector; MdNAC4-Anti: with MdNAC4 antisense vector. The WT expression level was set at 1. Error bars indicate the SDs of the three technical replicates and three biological replicates. Asterisks indicate significant differences between two independent samples according to t tests (*, *P* < 0.05 and **, *P* < 0.01)

To determine whether *MdNAC4* affects endogenous ABA production, transgenic apple calli were obtained (Fig. S1). First, we evaluated the ABA level in 2-week-old MdNAC4-OX, MdNAC4-Anti, and WT apple calli. The ABA content of MdNAC4-OX was 0.91 μg·g^− 1^ FW, which was significantly higher than that of WT apple calli (0.76 μg·g^− 1^ FW). The results suggest that the expression of ABA metabolic pathway genes may be affected by MdNAC4. qRT–PCR analysis of the expression levels of key ABA metabolic pathway genes showed that the expression of ABA biosynthetic genes, including *MdZEP*, *MdNCED1*, *MdNCED2* and *MdNCED6*, was significantly upregulated in MdNAC4-OX but that the expression of the ABA catabolism gene *MdCYP707A1* was downregulated (Fig. 2). Therefore, MdNAC4 may increase the level of endogenous ABA by upregulating ABA biosynthesis genes and downregulating ABA catabolic genes.

### MdNAC4 upregulates the expression of *MdNCED2*

**Fig. 3:**
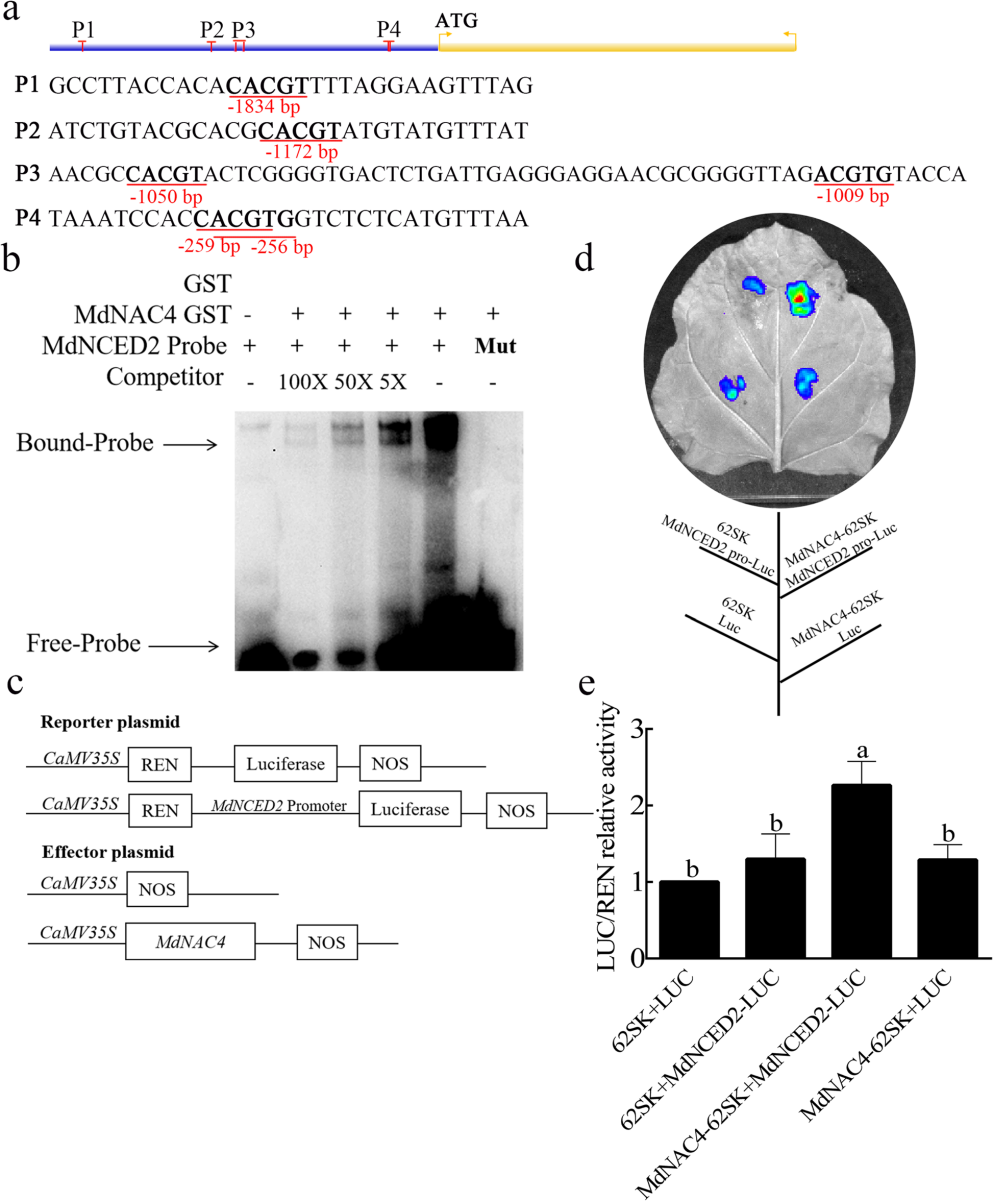
MdNAC4 activates the expression of *MdNCED2*. **a** Diagram of the *MdNCED2* gene promoter region. P1-P4 represent the potential sites to which MdNAC4 might bind. **b** The electrophoretic mobility shift assay (EMSA) showed that the MdNAC4-GST fusion protein was bound to the *MdNCED2* promoter. 5x, 50x and 100x represent the competitor concentrations. The unlabeled probes were used as competitors, with “Mut” representing the mutated probe in which the 5′-ACGTG-3′ motif was replaced by 5′-CCGTC-3′. **c** Structures of the reporter and effector vectors used in the dual-luciferase assays. The promoter fragment of *MdNCED2* was fused into the pGreenII 0800-LUC vector to obtain the reporter plasmid. The *MdNAC4* gene was fused to the pGreenII 62-SK vector to generate the effector plasmid. **d** Dual luciferase assays of tobacco leaves showed that MdNAC4 activated the expression of *MdNCED2*. **e** Relative LUC/REN activity analysis verified that MdNAC4 activated the expression of *MdNCED2*. Tobacco injected with empty vector was used as the control. Error bars indicate the SDs of the three technical replicates and three biological replicates. Different letters above the bars indicate significant differences according to one-way ANOVA (*P* < 0.05)

Previous studies have shown that NAC TFs can directly regulate the expression of *NCED3* and *ZEP* to participate in ABA signal transduction pathways (Mao et al. 2017; Sakuraba et al. 2020). To investigate whether MdNAC4 can also directly regulate the expression of the *NCED3* and *ZEP* genes. We first identified *NCED3* and *ZEP* genes in apple and then performed an analysis of promoter cis-acting elements. The apple homolog of *NCED3* is named *MdNCED2*, and six ABRE cis-acting elements were found in the promoter region 2 kb upstream of the start codon, which was divided into four regions according to distance (Fig. 3a). Apple *ZEP* is named *MdZEP*, but no ABRE cis-acting elements were found in its promoter region. To verify that MdNAC4 can bind to the ABRE (5-ACGTG-3) cis-acting element of the *MdNCED2* promoter, we performed EMSAs in vitro*.* As shown in Fig. S2, the MdNAC4-GST fusion protein could bind to the ABRE cis-acting element. According to the observed binding strength, we used P4 as the binding probe. In addition, the MdNAC4-GST fusion protein strongly bound to the *MdNCED2* probe but not to the mutant probe, and this binding gradually disappeared with an increasing competitor probe concentration (Fig. 3b).

Furthermore, to assess the positive regulation of *MdNCED2* expression by MdNAC4, we performed a dual luciferase assay with MdNAC4 as the effector and the luciferase gene under the control of MdNCED2 as the reporter (Fig. 3c, d). The results showed that the coexpression of MdNAC4 and the *MdNCED2* promoter significantly increased luciferase activity in tobacco leaves (Fig. 3e). In conclusion, the above data indicate that MdNAC4 directly binds to the promoter of *MdNCED2* and upregulates its expression.

### Overexpression of *MdNAC4* promotes N deficiency-induced senescence in tobacco leaves

**Fig. 4:**
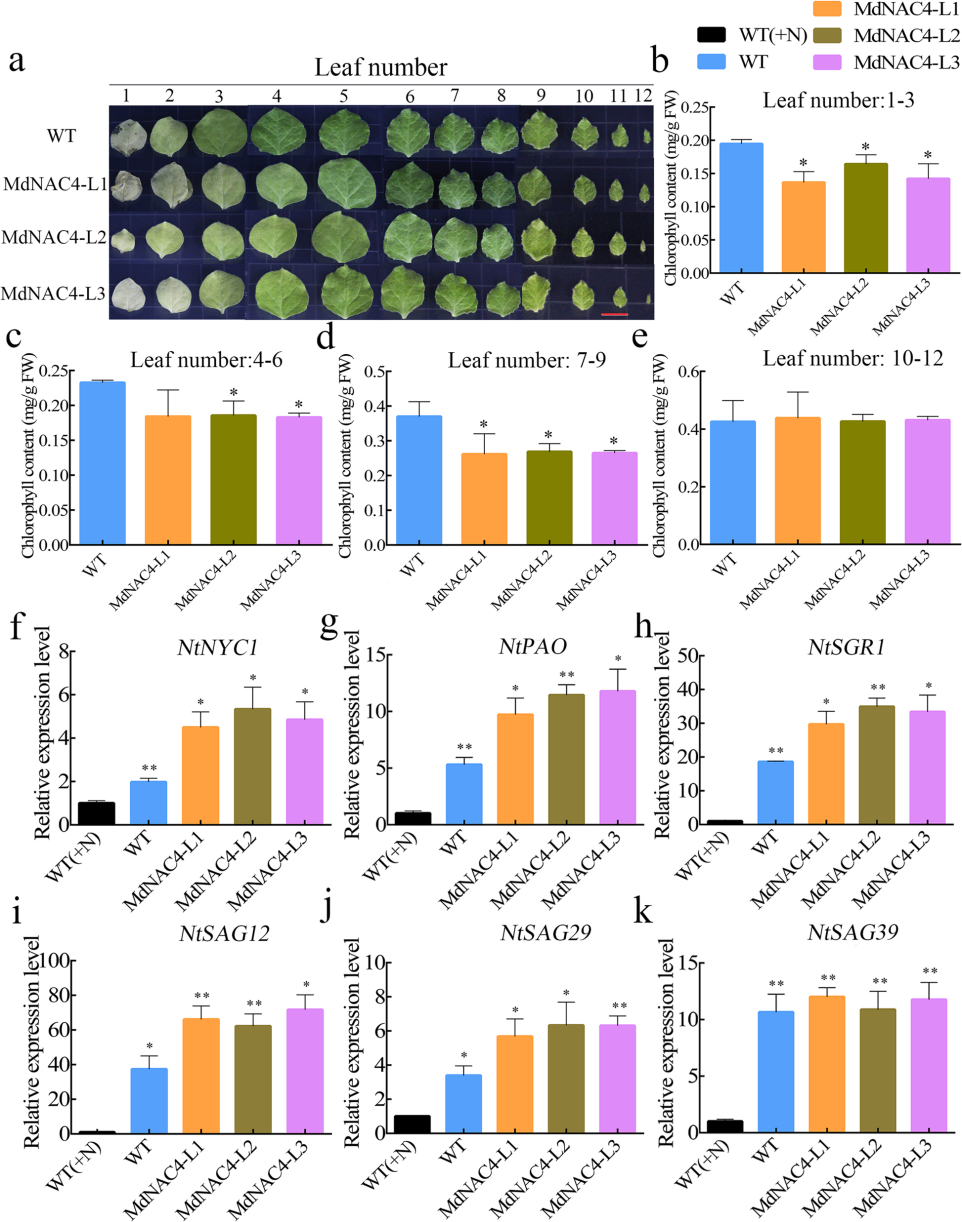
MdNAC4 caused early senescence induced by N deficiency in tobacco. **a** Leaf phenotypes of 4-week-old wild-type (Control) and transgenic tobacco (MdNAC4-L1, MdNAC4-L2 and MdNAC4-L3) grown in nitrate-deficient Hoagland nutrient solution for 3 weeks. Tobacco seedlings grown in Hoagland nutrient solution supplied with nitrate before treatment. Representative photographs were taken, where 1-12 represent the numbered leaf positions from the base to tip of tobacco leaves. **b-e** Total chlorophyll contents of tobacco leaves numbered 1-3, 4-6, 7-9, and 10-16 from 12 plants per indicated genotype. **f-k** Expression levels of *NtNYC1*, *NtPAO*, *NtSGR1*, *NtSAG12*, *NtSAG29* and *NtSAG39* in N-deficient wild-type and *MdNAC4-*overexpressing tobacco plants for 3 weeks. The expression level in the WT supplied with nitrate (+N) was set at 1. Error bars indicate the SDs of the three technical replicates and three biological replicates. Asterisks indicate significant differences between two independent samples according to t tests (*, *P* < 0.05 and **, *P* < 0.01)

To study the role of MdNAC4 in N deficiency-induced leaf senescence, three independent tobacco lines overexpressing *MdNAC4* (MdNAC4-L1, MdNAC4-L2 and MdNAC4-L3) were generated (Fig. S3). After 4-week-old tobacco seedlings were transferred to nitrate-deficient conditions for 3 weeks, *MdNAC4-*overexpressing transgenic tobacco showed a more severe senescence phenotype than the WT control (Fig. 4a). To explore the relationship between N deficiency and the *MdNAC4* gene, we examined the expression level of *MdNAC4* in tobacco under N-deficient conditions. Interestingly, the expression level of *MdNAC4* in transgenic tobacco under N-deficient conditions was significantly higher than that under N-sufficient conditions (Fig. S3b). In addition, the chlorophyll contents of leaves 1-3, 4-6, and 7-9 in transgenic tobacco were significantly lower than those in the WT (Fig. 4b-e), and the expression levels of senescence and chlorophyll catabolism-related genes (*NtNYC1*, *NtPAO*, *NtSGR1*, *NtSAG12*, *NtSAG29* and *NtSAG39*) were significantly higher than those in the WT (Fig. 4f-k). These results suggest that MdNAC4 is a positive regulator of N deficiency-induced leaf senescence and that its expression may depend on N-deficient conditions.

### MdNAC4 positively regulates the expression of senescence-related genes

**Fig. 5:**
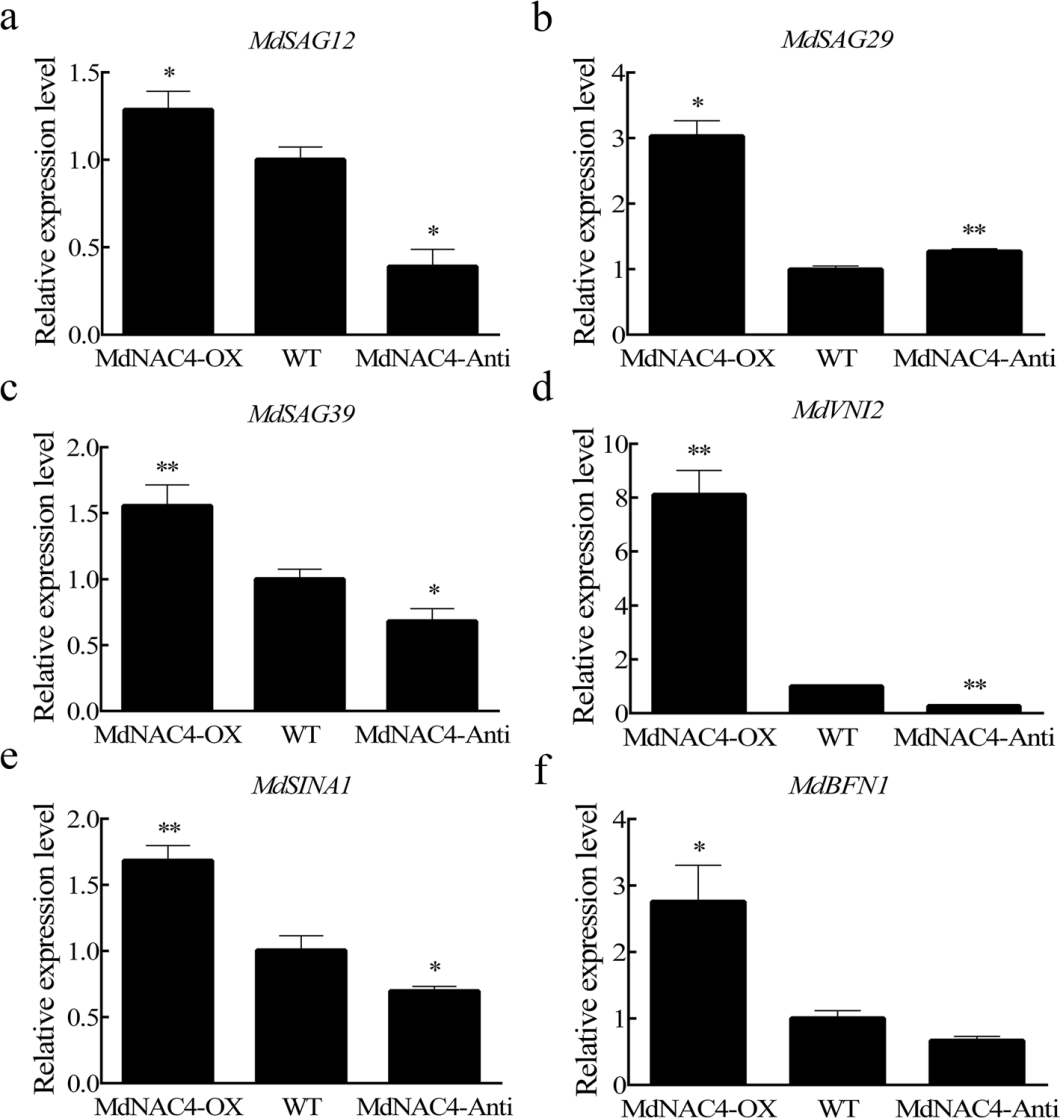
Expression levels of senescence-related genes (**a-f ***MdSAG12*, *MdSAG29*, *MdSAG39*, *MdVNI2*, *MdSINA1*, and *MdBFN1*) in 2-week-old apple calli. WT: wild-type; MdNAC4-OX: with *MdNAC4* overexpression vector; MdNAC4-Anti: with *MdNAC4* antisense vector. The WT expression level was set at 1. Error bars indicate the SDs of the three technical replicates and three biological replicates. Asterisks indicate significant differences between two independent samples according to t tests (*, P < 0.05 and **, P < 0.01)

As the expression levels of senescence-related genes are often used as markers of the senescence process, we examined the expression levels of senescence-related genes (*MdSAG12*, *MdSAG29*, *MdSAG39*, *MdVNI2*, *MdSINA1*, and *MdBFN1*) in 2-week-old transgenic (MdNAC4-OX and MdNAC4-Anti) and WT apple calli. As shown in Fig. 5, the expression of senescence-related genes was upregulated in MdNAC4-OX calli and downregulated in MdNAC4-Anti calli. These results indicate that MdNAC4 may accelerate the senescence process in apple calli by upregulating the expression of senescence-related genes.

### MdNAC4 upregulates the expression of *MdSAG39*

**Fig. 6:**
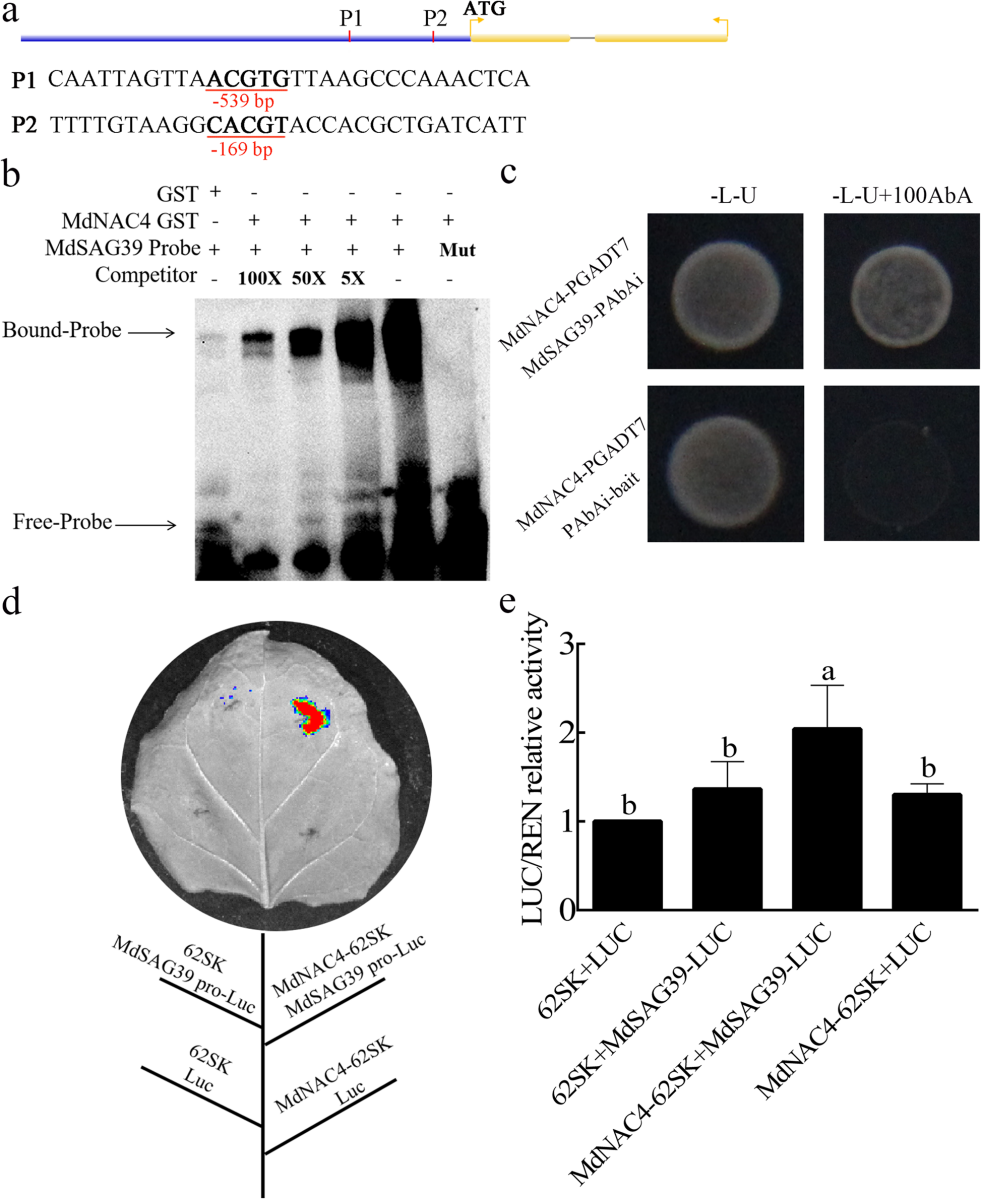
MdNAC4 activates the expression of *MdSAG39*. **a** Diagram of the *MdSAG39* gene promoter region. P1 and P2 represent the potential sites to which MdNAC4 might bind. **b** The electrophoretic mobility shift assay (EMSA) showed that the MdNAC4-GST fusion protein bound to the *MdSAG39* promoter. 5x, 50x and 100x represent the competitor concentrations. Unlabeled probes were used as competitors, with “Mut” representing the mutated probe in which the 5′-ACGTG-3′ motif was replaced by 5′-CCGTC − 3′. **c** A yeast one-hybrid (Y1H) assay revealed the interaction between MdNAC4 and the *MdSAG39* promoter. The cotransformed yeast strains were grown on SD/−L-U and SD/−L-U + 100 mM AbA medium for 3 days. **d** Dual luciferase assays of tobacco leaves showed that MdNAC4 activated the expression of *MdSAG39*. **e** Relative LUC/REN activity analysis verified that MdNAC4 activated the expression of *MdNCED2*. Tobacco injected with the empty vector was used as the control. Error bars indicate the SDs of the three technical replicates and three biological replicates. Different letters above the bars indicate significant differences according to one-way ANOVA (*P* < 0.05)

Previous studies have shown that *SAG39* can respond to leaf senescence signals and participate in the leaf senescence process (Liu et al. 2010). In addition, NAC TFs can regulate the expression of SAGs to accelerate leaf senescence. Therefore, the SAG *SAG39* may be a target gene of NAC TFs. To explore this possibility, we first analyzed the promoter sequence of *MdSAG39*. Two ABRE (5-ACGTG-3) cis-acting elements specifically bound by NAC TFs were found in the promoter region 2 kb upstream of the start codon of *MdSAG39* (Fig. 6a). To verify that MdNAC4 can directly bind to this ABRE cis-acting element, in vitro and in vivo experiments were performed based on EMSAs and yeast one-hybrid assays, respectively. As depicted in Fig. S4, two conserved motifs were identified as putative MdNAC4-GST fusion protein-binding sites. According to the observed binding strength, we used P2 as the binding probe. In addition, the MdNAC4-GST fusion protein bound specifically to the *MdSAG39* probe (ABRE 5-ACGTG-3) but not to the mutated probe (5-CCGTC-3). Furthermore, binding disappeared under increasing concentrations of competitor probe (Fig. 6b). In Y1H assays, yeast strains containing the pGADT7-MdNAC4 and pAbAi-MdSAG39 fusion plasmids grew normally on the selection medium, whereas the growth of the yeast strain containing the pGADT7 empty vector and the pAbAi-MdSAG39 fusion plasmid was inhibited on the screening medium (Fig. 6c).

Moreover, a dual effector–reporter system was constructed with MdNAC4 as the effector and the MdSAG39 promoter as the reporter, and only the coexistence of MdNAC4-62SK and MdSAG39 pro-LUC induced high luciferase activity in tobacco leaves (Fig. 6d, e). These results demonstrate that MdNAC4 directly binds to the promoter of the senescence-related gene *MdSAG39* and activates its expression.

### ABA enhances the function of MdNAC4 in leaf senescence induced by N deficiency

**Fig. 7:**
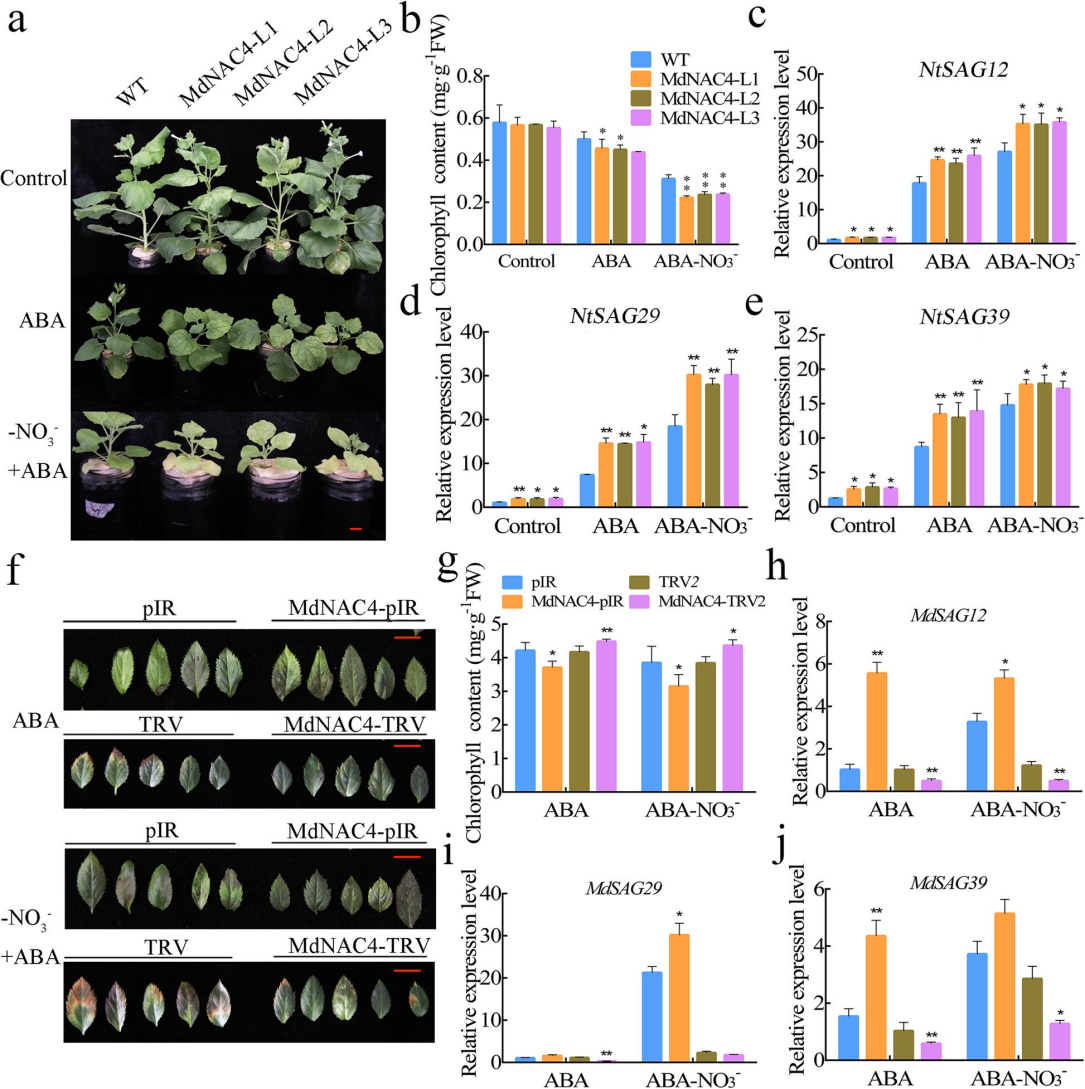
ABA enhances the senescence symptoms of  *MdNAC4* transgenic tobacco and apple leaves under N deficiency conditions

Considering that *MdNAC4* is responsive to ABA treatment and that MdNAC4 promotes N deficiency-induced leaf senescence, we investigated whether MdNAC4 is involved in ABA-mediated leaf senescence. First, 4-week-old tobacco seedlings were incubated with 30 μM ABA for 3 weeks. The growth of WT control tobacco leaves was inhibited, and the leaves showed a senescence phenotype, which seemed to be enhanced by the presence of overexpressed *MdNAC4*. The senescence phenotype of tobacco leaves became more pronounced under N-deficiency and ABA treatments (Fig. 7a). Accordingly, the decrease in chlorophyll content displayed a similar trend associated with the process of leaf senescence (Fig. 7b). Furthermore, we examined the transcript levels of *MdNAC4* in ABA- and -NO_3_^−^ + ABA-treated tobacco. The results showed that the transcript level of *MdNAC4* was significantly increased in ABA-treated tobacco and further increased in -NO_3_^−^ + ABA-treated tobacco (Fig. S5). We also detected the expression levels of the senescence-related genes *NtSAG12*, *NtSAG29* and *NtSAG39* in these seedlings by qRT–PCR and found the three genes to be significantly induced by the overexpression of *MdbNAC4* or ABA treatment alone; the expression of these three genes was further induced in the presence of both *MdNAC4* overexpression and ABA under N deficiency (Fig. 7c-e). Similarly, the overexpression of *MdNAC4* in apple leaves led to a severe senescence phenotype and lower chlorophyll content, whereas the antisense expression of *MdNAC4* caused a weaker senescence phenotype and higher chlorophyll content (Fig. 7f, g). qRT–PCR results also indicated that the senescence-related genes *MdSAG12*, *MdSAG29* and *MdSAG39* were significantly induced by either *MdNAC4* overexpression or ABA treatment alone and that the expression of these three genes was further induced when *MdNAC4* was overexpressed in the presence of ABA and N deficiency (Fig. 7h-j). These results suggest that ABA enhances the function of MdNAC4 in leaf senescence induced by N deficiency.

### The MdNAC4 protein interacts with the MdPYL4 protein

**Fig. 8:**
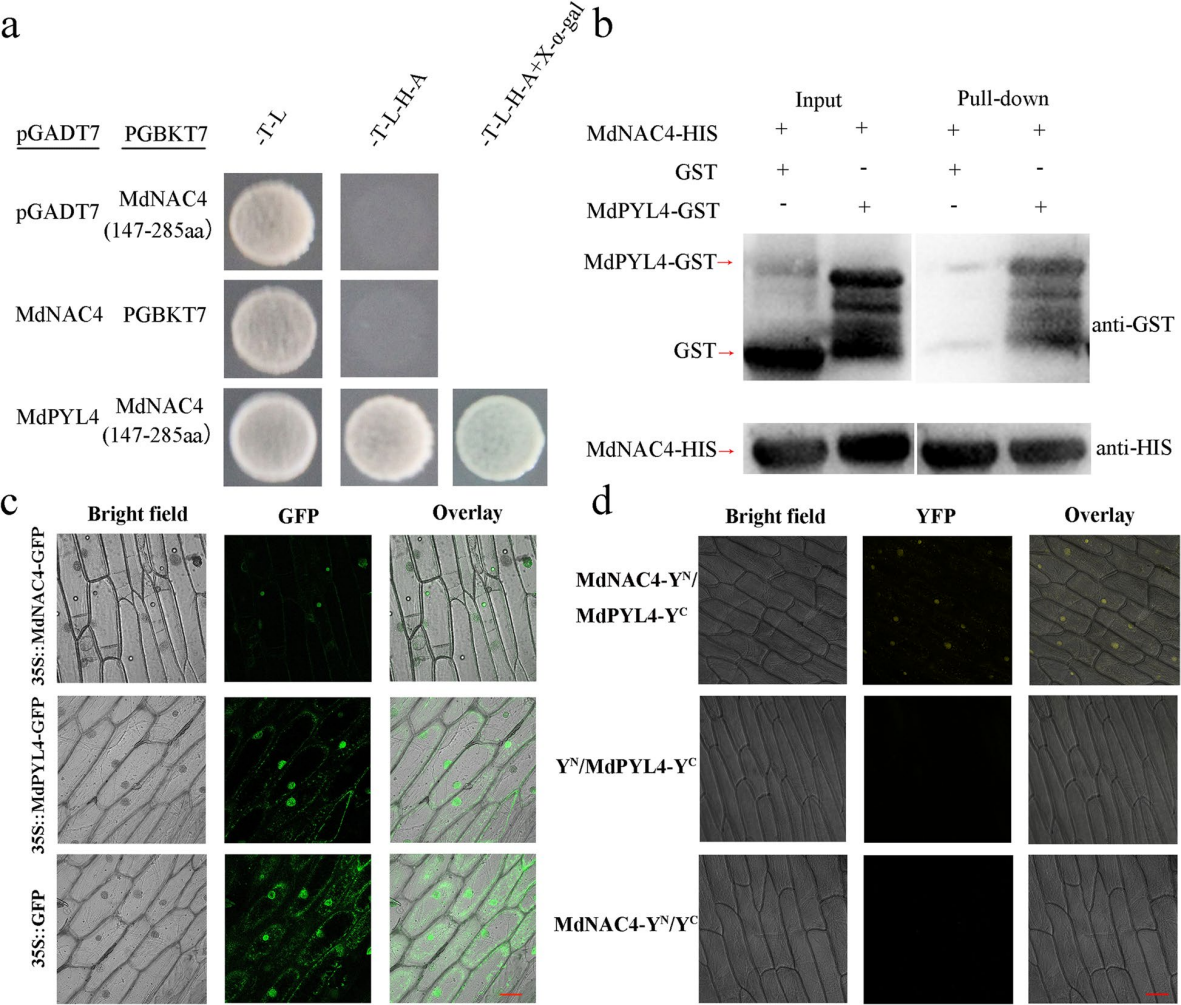
MdNAC4 interacts with MdPYL4. **a** Interaction between MdNAC4 and MdPYL4 in the Y2H assay. Full-length MdPYL4 was fused into the pGADT7 vector to obtain a recombinant plasmid (pGADT7-MdPYL4). The recombinant plasmid pGBKT7-MdNAC4 was obtained by fusing the MdNAC4 fragment without the autonomous activation domain into the pGBKT7 vector. Empty pGADT7 + pGBKT7-MdNAC4 (147-285 aa) and empty pGBKT7 + pGADT7-MdNAC4 were used as controls. The cotransformed yeast grown in SD (−T/−L), SD (−T/−L/−H/−A) and SD (−T/−L/−H/−A + X-α-gal) medium are indicated. **b** Interaction between the MdNAC4 and MdPYL4 proteins in the pull-down assay. The glutathione transferase (GST), MdPYL4-GST and MdNAC4-HIS proteins were induced by isopropyl thiogalactoside in *Escherichia coli.* The GST and MdPYL4-GST proteins were incubated with the MdNAC4-HIS protein, and the protein mixture was purified using the GST purification kit. **c** Subcellular localization of the MdPYL4 protein and the MdNAC4 protein. GFP, green fluorescent protein. **d** BiFC assays showed that the MdNAC4 protein interacted with the MdPYL4 protein. YFP, yellow fluorescent protein

To further explore the function of MdNAC4, we performed a yeast two-hybrid assay to screen the interacting proteins of MdNAC4. Based on previous studies, we used pGBKT7-MdNAC4 (147-285 aa) as bait (Wen et al. 2022). The results identified the ABA receptor protein MdPYL4 as an interacting protein of MdNAC4. To verify the interaction between the MdPYL4 and MdNAC4 proteins, the full-length CDS of MdPYL4 was inserted into the pGADT7 vector, and the 147-285 aa region of MdNAC4 was inserted into the pGBKT7 vector. The recombinant plasmids pGADT7-MdPYL4 and pGBKT7-MdNAC4 (147-285 aa) were cotransformed into Y2H yeast strains for yeast two-hybrid assays. Only yeast strains carrying both MdPYL4 and MdNAC4 (147-285 aa) were able to grow on SD/−T/−L/−H/−A medium and turned blue in the presence of X-α-gal, whereas the control did not (Fig. 8a). These results indicate that MdPYL4 interacts with MdNAC4 in vivo.

In addition, the fusion proteins MdPYL4-GST and MdNAC4-HIS were generated in *E. coli*, and a pull-down assay was performed. The MdPYL4-GST protein was pulled down by MdNAC4-HIS, but the control protein was not (Fig. 8b), indicating that MdPYL4 interacts with MdNAC4 in vitro.

Finally, we verified the interaction between MdPYL4 and the MdNAC4 protein in a BiFC assay. To determine the interacting regions of the MdPYL4 protein and MdNAC4 protein, we performed a subcellular localization analysis of these two proteins. We constructed the 35S::MdPYL4-GFP and 35S::MdPYL4-GFP plasmids and transformed onion epidermal cells with the empty 35S::GFP vector as a control. The fluorescence detection results indicated that the MdPYL4 protein localized to the nucleus and plasma membrane, while the MdNAC4 protein was localized only in the nucleus (Fig. 8c). Additionally, the MdPYL4 protein was fused to the pSPYCE (YFP^C^) vector, and the MdNAC4 protein was fused to the pSPYNE (YFP^N^) vector. The recombinant MdPYL4-YFP^C^ and MdNAC4-YFP^N^ plasmids were used to infect onion epidermal cells, and the fluorescence detection results showed a YFP signal only in cotransfected onion epidermal cells (Fig. 8d). Furthermore, the MdPYL4-MdNAC4 interacting protein complex appeared to be located in the nucleus. Taken together, these results indicate that the MdPYL4 protein interacts with the MdNAC4 protein in vivo and in vitro.

### Overexpression of *MdPYL4* promotes ABA-induced leaf senescence

**Fig. 9:**
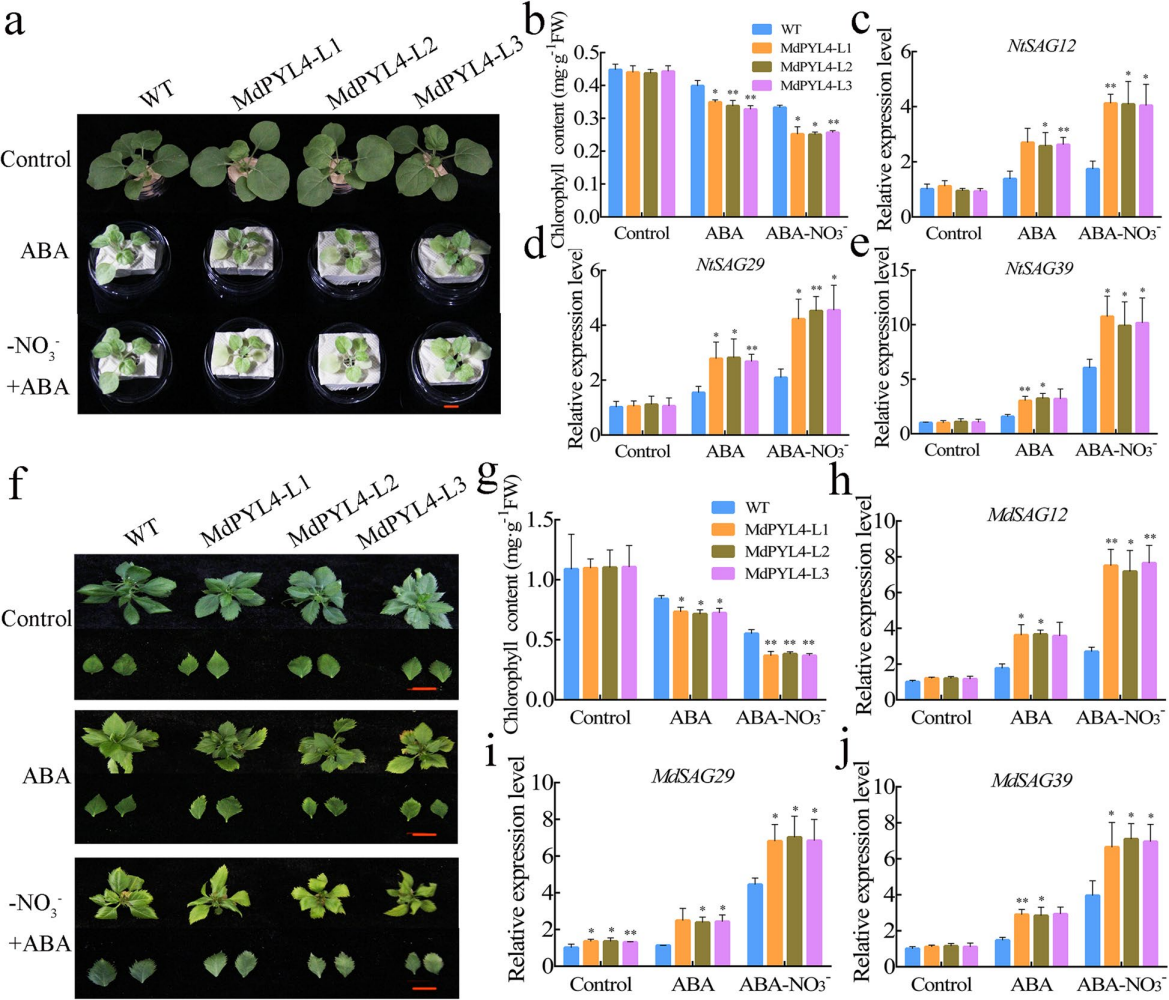
Overexpression of MdPYL4 promotes ABA-induced leaf senescence under N deficiency conditions

As both ABA and *MdNAC4* overexpression promote N deficiency-induced leaf senescence, MdPYL4 acts as a receptor for ABA and interacts with MdNAC4. Therefore, MdPYL4 may play an important role in ABA-mediated leaf senescence. To test the function of MdPYL4 in ABA-induced leaf senescence, we obtained tobacco and apple seedlings overexpressing *MdPYL4* (Fig. S6). When four-week-old tobacco seedlings were transferred to conditions in the presence of ABA for 10 days, WT control leaves showed a senescence phenotype, which appeared to be enhanced by N deficiency or *MdPYL4* overexpression (Fig. 9a). Accordingly, chlorophyll degradation associated with leaf senescence showed a similar trend (Fig. 9b). In addition, the expression of the SAGs *NtSAG12*, *NtSAG29*, and *NtSAG39* was examined by qRT–PCR (Fig. 9c-e). In the presence of ABA, the expression of SAGs in *MdPYL4*-overexpressing tobacco was significantly higher than that in the WT control; the expression of SAGs was further increased under combined ABA and N deficiency. Similarly, in transgenic apple seedlings, the overexpression of *MdPYL4* caused similar senescence symptoms and a lower chlorophyll content when the plants were treated with ABA for 4 weeks. *MdPYL4*-overexpressing apple seedlings exhibited more severe senescence symptoms and a lower chlorophyll content under combined N deficiency and ABA treatment (Fig. 9f, g), and the expression of the SAGs *MdSAG12*, *MdSAG29* and *MdSAG39* showed a similar trend to leaf senescence (Fig. 9h-j). These results indicate that MdPYL4 plays a key role in ABA-induced leaf senescence and may promote the leaf senescence phenotype by enhancing the response to ABA by integrating N deficiency signals.

## Discussion

Leaf senescence is a highly coordinated developmental process. Its initiation is mainly regulated by the integration of a series of internal and external factors providing age-dependent information (Sakuraba et al. 2020; Lim et al. 2007). Plant hormones are considered to be important players in leaf senescence and can affect each stage of the process. In addition, plant hormones can integrate environmental signals into the process of plant development, thus altering leaf senescence (Lee and Masclaux-Daubresse 2021). Nevertheless, the effect of environmental factors on leaf senescence is not independent but involves mutual promotion or inhibition (Guo and Gan 2012). Thus, interactions between plant hormones, developmental processes and environmental factors may determine the onset of leaf senescence. Environmental stress signals stimulate changes in endogenous hormone contents, integrate them into the process of plant development, and then affect leaf senescence through complex regulatory networks.

The senescence of leaves is accompanied by changes in the expression of hundreds of SAGs (Kim et al. 2016). To date, a total of 5853 SAGs and 617 mutants associated with leaf senescence have been identified in 68 species (Li et al. 2020). Among the identified SAGs, NAC TFs play important roles in modulating the leaf senescence process by regulating gene expression. Previous studies have shown that the NAC transcription factor NAC2 upregulates ABA biosynthesis genes (*NCED3* and *ZEP1*) and modulates the expression of chlorophyll catabolism genes (*SGR* and *NYC3*) to promote leaf senescence (Mao et al. 2017). NAC096 upregulates the ABA signaling gene *ABI5* to mediate ABA-induced leaf senescence (Kang et al. 2019). NAC054 directly activates the expression of the ABA signaling gene *ABI5* and the chlorophyll catabolism gene *NYC1* to promote ABA-induced leaf senescence (Sakuraba et al. 2020). In addition, some NAC TFs (NAP, ANAC072, ANAC092, and ANAC109) positively regulate leaf senescence (Li et al. 2021; Liang et al. 2014; Wang et al. 2021; Park et al. 2018). Other NAC TFs (NAL, ANAC042, ANAC083, and ANAC106) appear to negatively regulate leaf senescence (Sakuraba et al. 2015; Wu et al. 2012; Yang et al. 2011; Yu et al. 2022).

ABA is considered one of the most effective plant hormones in promoting leaf senescence, and exogenous ABA promotes leaf senescence by inducing the expression of SAGs (Lee et al. 2011). In this study, we identified an NAC TF, MdNAC4, that positively regulates leaf senescence by regulating the expression of ABA metabolism genes. Furthermore, we found that the ABA content was higher in MdNAC4-OX apple calli and lower in MdNAC4-Anti calli than in WT calli. Consistent with this, the expression of ABA biosynthesis genes was upregulated, but that of ABA catabolism genes was downregulated in MdNAC4-OX calli (Fig. 2). Previous studies have shown that 9-cis-epoxycarotenoid dioxygenase (NCED) catalysis and ABA 8′-hydroxylase-mediated hydroxylation are key steps regulating the level of endogenous ABA (Nambara and Marion-Poll 2005). Our results provide evidence that the activity of 9-cis-epoxycarotenoid dioxygenase 2 (NCED2) is critical for regulating ABA levels (Fig. 3). In rice, OsNCED3 is involved in the cleavage of xanthophyll, and *nced3* mutant leaves exhibit a stay-green phenotype (Hwang et al. 2010; Mao et al. 2017). It is possible that the mutation of *NCED3* leads to the inhibition of ABA biosynthesis, which delays leaf senescence. Furthermore, MdNAC4 induces the expression of *NCED2*, which suggests that *NCED2* acts downstream of *MdNAC4*. These results suggest that MdNAC4 promotes leaf senescence-dependent ABA biosynthesis in apple.

Nutrient deficiency is an important environmental factor that induces leaf senescence, and deficiency of any nutrient can cause early leaf senescence (Sade et al. 2018; Guo and Gan 2012). N is an essential element for plant growth and development, and its deficiency tends to induce rapid leaf senescence (Park et al. 2018). Our previous studies have shown that the NAC TF MdNAC4 promotes N deficiency-induced leaf senescence by regulating the expression of the CCGs *MdNYC1* and *MdPAO* (Wen et al. 2022). In this study, we found that MdNAC4 promotes N deficiency-induced leaf senescence by activating the ABA biosynthetic gene *MdNCED2* to increase ABA content. Therefore, ABA may be a key phytohormone involved in the regulation of N deficiency-induced leaf senescence by MdNAC4. Leaf senescence is a developmental process regulated by SAGs. The functional identification and regulatory network analysis of SAGs provide insight into the molecular mechanism of leaf senescence. Previous studies have shown that NAC TFs are involved in leaf senescence by regulating the expression of SAGs. For example, the *Arabidopsis* NAC TF NAP regulates leaf senescence by directly binding to the promoter of the *SAG113* gene (Zhang and Gan 2012). Additionally, ANAC032 alters the expression of *SAG113* to positively regulate stress-induced leaf senescence (Mahmood et al. 2016), and ANAC092 promotes leaf senescence by upregulating the expression of the senescence-related gene *SAG29* (Matallana-Ramirez et al. 2013). Our current studies indicate that MdNAC4 can directly activate the transcription of the SAG *MdSAG39* to regulate N deficiency-induced leaf senescence*.* Furthermore, in *MdNAC4* transgenic tobacco, the expression level of MdNAC4 was found to be approximately 10-fold higher under N-deficient conditions than under N-sufficient conditions (Fig. S3b). Therefore, MdNAC4 may directly regulate N deficiency-induced leaf senescence independent of other pathways. In addition, MdNAC4 could accelerate N deficiency-induced leaf senescence by activating the transcription of ABA biosynthesis pathway genes to increase ABA content.

It is well established that N deficiency induces leaf senescence, but how N deficiency interacts with internal plant signals remains largely unknown. Plant hormones are important factors that affect leaf senescence, integrate environmental signals and regulate leaf senescence through a complex signaling network (Wen et al. 2020). Under salt stress conditions, the NAC transcription factor VNI2 integrates ABA signaling in leaf senescence (Yang et al. 2011). Under drought stress conditions, ABA regulates leaf senescence by activating sucrose nonfermenting 1-related protein kinase 2 s (SnRK2s) (Zhao et al. 2016). Under dark treatment, NAP promotes leaf senescence by enhancing the transcription of the ABA biosynthesis gene *AAO3* (Yang et al. 2014). These results indicate that leaf senescence caused by various abiotic stresses may be achieved through ABA signaling. In this study, we found that exogenous ABA enhances the function of MdNAC4 in N deficiency-induced leaf senescence (Fig. 7). Furthermore, ABA signaling analysis showed that ABA-promoted leaf senescence is mediated by three key components of the pathway (PYLs–PP2C–SnRK2) (Gao et al. 2016). Thus, PYL family members play a key role in ABA-mediated leaf senescence. We also identified an ABA receptor protein, MdPYL4, that interacts with the MdNAC4 protein (Fig. 8). The subcellular localization analysis of the MdPYL4 protein showed that the MdPYL4 protein was located in the nucleus and plasma membrane. However, the MdPYL4-MdNAC4 interacting protein complex was mainly located in the nucleus. Additionally, previous studies showed that the PP2CA-PYL4 protein-interacting complex localized to the cytosol (Pizzio et al. 2013), the RING FINGER OF SEED LONGEVITY1 (RSL1)-PYL4 protein-interacting complex localized to the plasma membrane (Bueso et al. 2014), and the ABA INSENSITIVE 2 (ABI2)-PYL4 protein-interacting complex localized to both the cytoplasm and nucleus (Wang et al. 2020). These results suggested that the localization of proteins interacting with the PYL4 protein affected the localization of the interaction complex. In this study, the MdPYL4 protein-interacting protein MdNAC4 was found to localize to the nucleus. Additionally, MdNAC4 increased ABA contents by upregulating the expression of the ABA biosynthesis-related gene *MdNCED2*. ABA enhanced the relative fluorescence of the MdPYL4-MdNAC4 complex in the nucleus. Thus, MdPYL4 may function in an ABA-dependent interaction with MdNAC4 to promote the initiation of leaf senescence. In addition, studies on *Arabidopsis* have revealed that ABA promotes leaf senescence through ABA receptors (Zhao et al. 2016). Here, we found that the overexpression of *MdPYL4* promoted ABA-induced leaf senescence and that N deficiency enhanced the senescence phenotype (Fig. 9). These observations suggest that the interaction between the ABA receptor protein MdPYL4 and the MdNAC4 protein may enhance the response of MdNAC4 to N deficiency, which may be the key process by which MdNAC4 promotes N deficiency-induced leaf senescence.

**Fig. 10:**
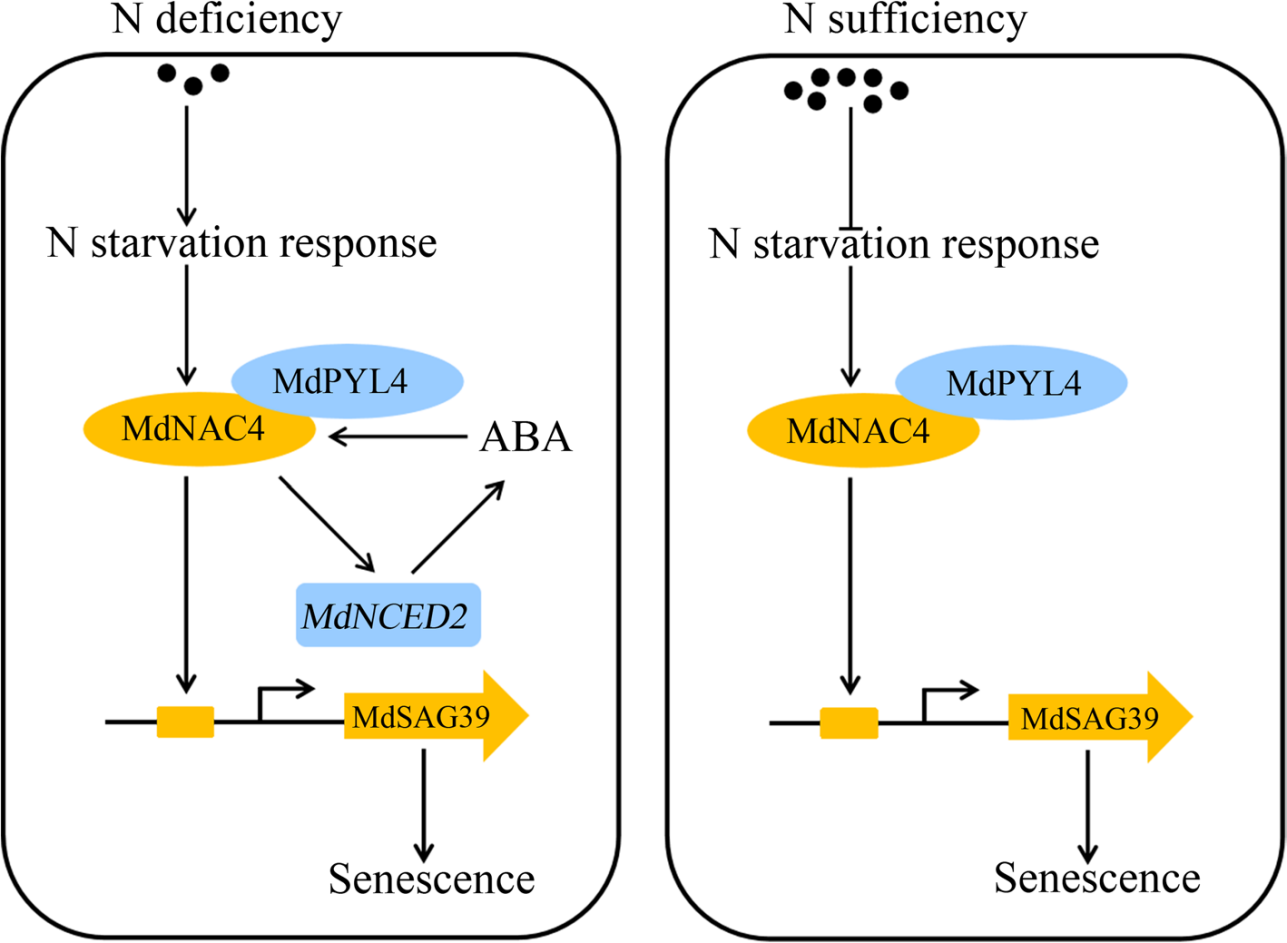
A working model of the MdNAC4-mediated regulation of N deficiency-induced leaf senescence in apple. Under N-deficient conditions, N starvation-responsive genes are activated, which induces the expression of *MdNAC4*. MdNAC4 directly activates the expression of the ABA biosynthesis gene *MdNCED2* and further promotes ABA biosynthesis. The ABA receptor protein MdPYL4 interacts with the MdNAC4 protein and enhances the response of *MdNAC4* to N deficiency. MdNAC4 directly binds to the SAG *MdSAG39* and activates its expression, thus promoting leaf senescence induced by N deficiency. Under N-sufficient conditions, N starvation-responsive genes are suppressed. MdPYL4 interacts with MdNAC4 proteins to activate the expression of SAG *MdSAG39*, thus regulating leaf senescence

In conclusion, based on previous studies and our findings, a working model of N deficiency-induced leaf senescence is proposed (Fig. 10). Under N-deficient conditions, MdNAC4 directly activates the transcription of the ABA biosynthesis gene *MdNCED2*, thus increasing ABA levels. ABA further induces the expression of MdNAC4 to form a feedback loop. Moreover, MdNAC4 directly binds to the promoter of the SAG *MdSAG39* and activates its expression to promote leaf senescence induced by N deficiency. The presence of ABA enhances the function of MdNAC4 in leaf senescence induced by N deficiency. In addition, the ABA receptor protein MdPYL4 interacts with the MdNAC4 protein to enhance the response of *MdNAC4* to N deficiency, which promotes N deficiency-induced leaf senescence. Our results provide new insight into the metabolic pathway of leaf senescence induced by N deficiency.

## Methods

### Plant materials, growth conditions, and treatments

The tissue culture apple seedlings used in this study were *Malus × domestica* ‘GL 3’ seedlings grown in MS medium containing 0.2 mg·L^− 1^ NAA, 0.6 mg·L^− 1^ 6-BA, and 0.2 mg·L^− 1^ GA_3_. ‘GL 3’ tissue culture seedlings were grown under long-day conditions at normal temperature (24 ± 1 °C 14 h light/10 h dark) and subcultured once a month. *Malus domestica* ‘Orin’ calli were grown in MS medium containing 1.5 mg·L^− 1^ 2,4-D and 0.4 mg·L^− 1^ 6-BA. The calli were grown in the dark at 24 °C and subcultured every 2 weeks. *Nicotiana benthamiana* seedlings were also used. For the tissue culture of tobacco seedlings, sterilized seeds were placed on 1/2 MS solid medium, incubated at 4 °C for 96 h and then transferred to an incubator set at 22 °C under long-day conditions (14 h light/10 h dark). For soil-grown tobacco seedlings, vernalized seeds were sown on the soil surface and cultivated under long-day conditions at 22 °C (14 h light/10 h dark).

For assays of nitrate deficiency-induced leaf senescence in tobacco seedlings, four-week-old tobacco seedlings were grown for 3 weeks in N-deficient modified Hoagland’s solution in which CaCl_2_ and KCl were used instead of Ca(NO_3_)_2_ and KNO_3_ to observe the senescence phenotype. For ABA-induced and combined N deficiency- and ABA-induced leaf senescence assays, detached leaves were placed on medium containing 50 μm ABA or nitrate-deficient medium containing 50 μm ABA at 22 °C under light to observe the senescence phenotype. For ABA-induced and combined N deficiency- and ABA-induced leaf senescence assays in tobacco seedlings, four-week-old tobacco seedlings were placed in Hoagland’s solution (505.50 mg/L KNO_3,_ 1180.80 mg/L Ca(NO3)_2_·4H_2_O, 492.90 mg/L MgSO_4_·7H_2_O, 136.10 mg/L KH_2_PO_4_, 2.86 mg/L H_3_BO_3_, 1.81 mg/L MnCl_2_·4H_2_O, 0.22 mg/L ZnSO_4_·7H_2_O, 0.08 mg/L CuSO_4_, 0.09 mg/L H_3_MoO_4_·H2O, 5.56 mg/L FeSO_4_·7H_2_O, 7.46 mg/L EDTA·Na_2_, Hoagland and Arnon 1950) containing 30 μm ABA or modified Hoagland’s solution containing 30 μm ABA in which CaCl_2_ and KCl were used instead of Ca(NO_3_)_2_ and KNO_3_ for 3 weeks to observe the senescence phenotype. For ABA-induced and combined N deficiency- and ABA-induced leaf senescence assays of apple seedlings, one-month-old apple seedlings were subcultured in medium containing 50 μm ABA or N-deficient medium (Murashige and Skoog’s solution containing 1401 mg/L KCl and 1103 mg/L NH_4_Cl (pH 5.8) instead of 1900 mg/L KNO_3_ and 1650 mg/L NH_4_NO_3_) containing 50 μm ABA for 4 weeks to observe the senescence phenotype.

### Plasmid construction and genetic transformation

The full-length sequences of the *MdNAC4* and *MdPYL4* genes were obtained from apple (https://www.rosaceae.org/) databases. Specific primers were designed according to the sequences for PCR amplification. The PCR products were inserted into the pBI121-GFP vector to construct overexpression plasmids under the control of the 35S promoter. The constructed recombinant plasmids were transformed into *Agrobacterium tumefaciens* strain LBA4404. *Agrobacterium*-mediated genetic transformation was used to obtain transgenic apple seedlings, calli and tobacco seedlings (Zhao et al. 2020; An et al. 2018). The cDNA fragment of MdNAC4 was amplified by RT–PCR to construct antisense expression vectors. The PCR products were inserted into the TRV vector under the control of the 35S promoter. The recombinant vector was named MdNAC4-TRV2. The MdNAC4-TRV2 recombinant plasmids were transformed into *Agrobacterium tumefaciens* strain LBA4404 for inoculation. The transient overexpression vector was constructed using the same method employed for the antisense expression vectors. The PCR products were inserted into IL60 vectors, and the recombinant vector was named MdNAC4-IL60. The MdNAC4-IL60 recombinant plasmids were transformed into *Agrobacterium tumefaciens* strain LBA4404 for inoculation. The tissue-cultured seedlings were grown for 1 month, and functional leaves showing the same amount of growth were selected for infection. The transient transformation of transgenic apple leaves was performed as described previously (An et al. 2019; Hu et al. 2016; Wang et al. 2022). The primers used for genetic transformation are provided in Table S1.

### Determination of chlorophyll contents

The chlorophyll contents of apple and tobacco leaves subjected to different treatments were determined as previously described (Wen et al. 2019). Chlorophyll was extracted from 0.2 g senescent leaves homogenized with 20 mL 96% ethanol. After extraction under dark conditions for 24 h, the absorbance at 470, 649 and 665 nm was measured using a spectrophotometer (UV-2600 Shimadzu, Shanghai, China).

### GUS staining and activity analysis

A 2000-bp promoter sequence upstream of the *MdNAC4* start codon was fused to the pCAMBIA1300 vector to obtain the *MdNAC4* promoter::GUS recombinant plasmid (MdNAC4^**pro**^**-**GUS). The MdNAC4pro-GUS fusion plasmid was transformed into *A. tumefaciens* strain LBA4404. Transgenic apple calli obtained by the Agrobacterium-mediated transformation method were plated on medium containing 50 μm ABA for 9 h; wild-type calli were used as the control. GUS staining was performed as previously described (Xi et al. 2012), and the transcription level of the GUS gene was measured by quantitative real-time polymerase chain reaction (qRT–PCR).

### ABA content determination

Two-week-old apple calli of MdNAC4-OX, wild type (WT), and MdNAC4-Anti were used to assess ABA contents. The extraction and quantitative evaluation of ABA were performed as previously described (Chen et al. 2012).

### RNA extraction and gene expression analysis

Total RNA was extracted according to the instructions of the RNA Prep Pure Plant Plus Kit (TIANGEN, Beijing, China). Single-strand cDNA was obtained by reverse transcription of RNA using a cDNA Synthesis Kit (Vazyme, Nanjing, China). Quantitative real-time polymerase chain reaction was performed as previously described (Wen et al. 2019). The analysis of gene expression levels was performed using the comparative Ct (2^-ΔΔCt^) method. Three technical and biological replicates were performed for each sample. The primers used for qRT–PCR are listed in Table S1.

### EMSAs

The *MdNAC4* coding sequence (CDS) was inserted into the PGEX4T-1 vector to obtain the MdNAC4-GST recombinant plasmid. The constructed recombinant plasmid was transformed into *Escherichia coli* BL21 cells (TransGen, Beijing, China), and protein expression was induced with 1 mm isopropyl-β-D-thiogalactopyranoside at 37 °C. Probe synthesis and biotin labeling were performed at Sangon Biotech Co., Ltd. (Shanghai, China). The fusion protein and biotin-labeled probes were incubated in light shift binding buffer for 30 minutes at 24 °C in the dark. The 5′-ACGTG-3′ sequence was replaced by the 5′-CCGTC-3′ sequence as the mutated probe, and an unlabeled probe was used for competition assays. The binding of the MdNAC4-GST fusion protein to the probe was detected by polyacrylamide gel electrophoresis (Thermo Scientific, San Jose, USA).

### Y1H assays

Yeast one-hybrid (Y1H) assays were performed as previously described (Zhao et al. 2021). Briefly, the *MdNAC4* CDS was fused to the pGADT7 vector. *MdSAG39* promoter fragments were fused to the pAbAi vector to generate the MdSAG39-pAbAi recombinant plasmid. The constructed MdSAG39-pAbAi plasmid was transformed into Y1H yeast, and aureobasidin A (AbA) concentration-suppressing pAbAi vector background expression was screened. Different combinations of the recombinant plasmids were cotransformed into Y1H yeast strains, and growth was observed on medium (SD/−Ura/−Leu) containing the selected concentration of AbA.

### Dual luciferase assays

Dual luciferase assays were performed as previously described (Hellens et al. 2005). *MdNCED2* and *MdSAG39* promoter fragments were fused to the pGreenII 0800-LUC vector to generate a reporter construct. The *MdNAC4* CDS was fused to pGreenII 62-SK to generate an effector construct. The constructed recombinant plasmids were transformed into *A. tumefaciens* strain GV3101, and tobacco leaves were infected with a mixture of reporter and effector *Agrobacterium* strains. LUC/REN activity was assessed using a dual-luciferase reporter assay system (Promega, Madison, USA).

### BiFC assays

The BiFC assay was performed as previously described (Chen et al. 2018). *MdNAC4* and *MdPYL4* CDSs were fused to the pSPYNE and pSPYCE vectors containing YFP fragments to generate the MdNAC4-YFP^N^ and MdPYL4-YFP^C^ constructs, respectively. The constructed recombinant plasmids were transformed into *Agrobacterium* strain GV3101 and used to infect onion epidermal cells with mixed *Agrobacterium* strains. The infected onion epidermal cells were transferred to MS medium and cultured at 28 °C in the dark for 1-2 days.

### Y2H assays

The *MdPYL4* CDS and the domain-deleted form (147-285 aa) of MdNAC4 were fused to the pGADT7 and pGBKT7 vectors to generate the pGADT7-MdPYL4 and pGBKT7-MdNAC4 ^147-285 aa^ constructs, respectively. Different combinations of the recombinant plasmids were cotransformed into Y2H yeast competent cells, and the empty vector was used as the control. Yeast transformants were cultured on SD/−Trp/−Leu medium at 30 °C for 3-5 days. Then, the putative transformants were transferred to SD/−Leu/−Trp/−His/−Ade selection medium and SD/−Leu/−Trp/−His/−Ade medium with X-α-gal.

### Pull-down assays

The *MdNAC4* and *MdPYL4* CDSs were fused to the pET32a and pGEX-4 T-1 vectors, respectively, and these two constructed recombinant plasmids were transformed into *E. coli* BL21 (TransGen, Beijing, China). The induction of HIS- and GST-tagged protein expression was achieved using 1 mm isopropyl-β-D-thiogalactopyranoside. After the incubation of MdNAC4-HIS with MdPYL4-GST or GST, pull-down assays were performed using a HIS-tagged protein purification kit (CW Biotech, Taizhou, China). The eluted proteins were separated and detected by immunoblotting using anti-HIS and anti-GST antibodies (Abmart, Shanghai, China).

### Statistical analysis

Statistical analysis was performed using SPSS 19 software (SPSS, Chicago, IL, USA) and GraphPad Prism 6 software (GraphPad Software, La Jolla, CA, USA). Significant differences between two independent samples were assessed by t tests (*, *P* < 0.05 and **, *P* < 0.01).

### Supplementary Information

**Additional file 1: Fig. S1.** Identification of transgenic apple calli.

**Additional file 2: Fig. S2.** MdNAC4 binds specific sequences in the *MdNCED2* promoter.

**Additional file 3: Fig. S3.** Identification of transgenic tobacco overexpressing *MdNAC4*.

**Additional file 4: Fig. S4.** MdNAC4 binds specific sequences of the *MdSAG39* promoter.

**Additional file 5: Fig. S5.** Expression level of *MdNAC4* in WT and *MdNAC4* transgenic tobacco plants after 30 μm ABA and -NO_3_^−^ + 30 μm ABA treatment.

**Additional file 6: Fig. S6.** Identification of transgenic tobacco and apple seedlings overexpressing *MdPYL4*.

**Additional file 7: Table S1.** The primers used in this study.

## Data Availability

All data supporting the findings of this study are included in the manuscript and its supplementary information.

## Abbreviations

N: Nitrogen
ABA: Abscisic acid
ETH: Ethylene
JA: Jasmonic acid
ACC: 1-aminocyclopropane-1-carboxylic acid
MeJA: Methyl jasmonate
NAC: NAM, ATAF1, ATAF2 and CUC2
TF: Transcription factor
SAG: Senescence-associated gene
AAO: Abscisic aldehyde oxidase
PYR/PYL: Pyrabactin resistance/pyr1-like
PP2C: Protein phosphatase 2C
SnRK: SNF1-related kinase
ABF: ABRE-binding factor
CCG: Chlorophyll catabolism-related gene
CLH: Chlorophyllase
NRT: Nitrate transporter
NCED: 9-cis-epoxycarotenoid dioxygenase
ZEP: Zeaxanthin epoxidase
NYC: Non-yellow coloring
PAO: Pheide a oxygenase
RSL: RING finger of seed longevity
ABI: ABA insensitive
GUS: Beta-glucuronidase
IPTG: Isopropyl-β-D-thiogalactopyranoside
Y1H: Yeast one-hybrid
AbA: Aureobasidin A
BiFC: Bimolecular fluorescence complementation
EMSA: Electrophoretic mobility shift assay
qRT-PCR: Quantitative real-time polymerase chain reaction
MS: Murashige & Skoog medium
