# Supplementary material for: The NAC transcription factor MdNAC4 positively regulates nitrogen deficiency-induced leaf senescence by enhancing ABA biosynthesis in apple
Source: Mol Hortic. 2023 Mar 10;3:5. doi: 10.1186/s43897-023-00053-4 (PMC10514974; doi:10.1186/s43897-023-00053-4)
Supplement: Supplementary file 1 — Additional file 1: Fig. S1. Identification of transgenic apple calli. [file 43897_2023_53_MOESM1_ESM.docx]

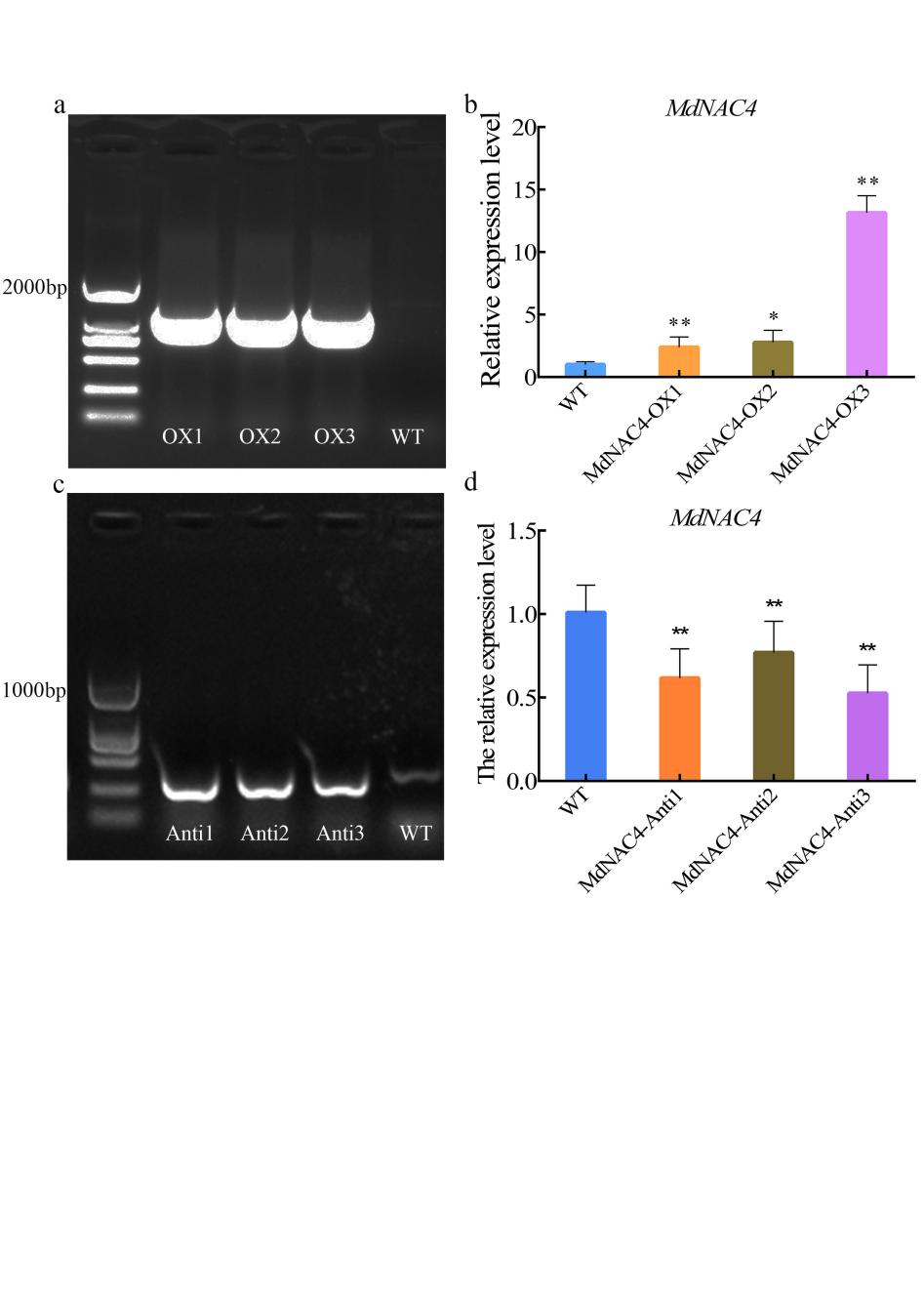


**Additional file 1: Fig. S1.** Identification of transgenic apple calli. Identification of transgenic apple calli with an *MdNAC4* overexpression construct (a) and an *MdNAC4* antisense construct (c) by PCR. Identification of transgenic apple calli with an *MdNAC4* overexpression construct (b) and an *MdNAC4* antisense construct (d) by qRT‒PCR. The WT expression level was set at 1. Error bars indicate the SDs of the three technical replicates and three biological replicates. Asterisks indicate significant differences between two independent samples according to t tests (*, P < 0.05 and **, P < 0.01).
