# Supplementary figures and images for: The NAC transcription factor MdNAC4 positively regulates nitrogen deficiency-induced leaf senescence by enhancing ABA biosynthesis in apple
Source: Mol Hortic. 2023 Mar 10;3:5. doi: 10.1186/s43897-023-00053-4 (PMC10514974; doi:10.1186/s43897-023-00053-4)

**
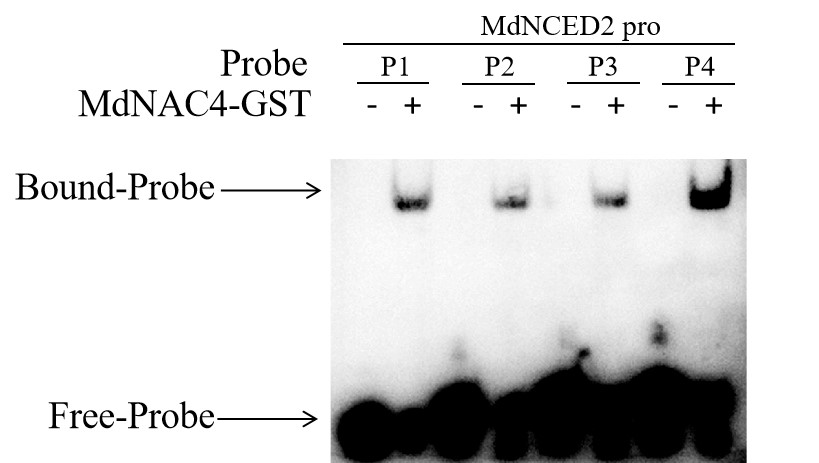
**

**Additional file 2: Fig. S2** MdNAC4 binds specific sequences in the *MdNCED2* promoter.

Supplement: Supplementary file 2 — Additional file 2: Fig. S2. MdNAC4 binds specific sequences in the MdNCED2 promoter. [file 43897_2023_53_MOESM2_ESM.docx]

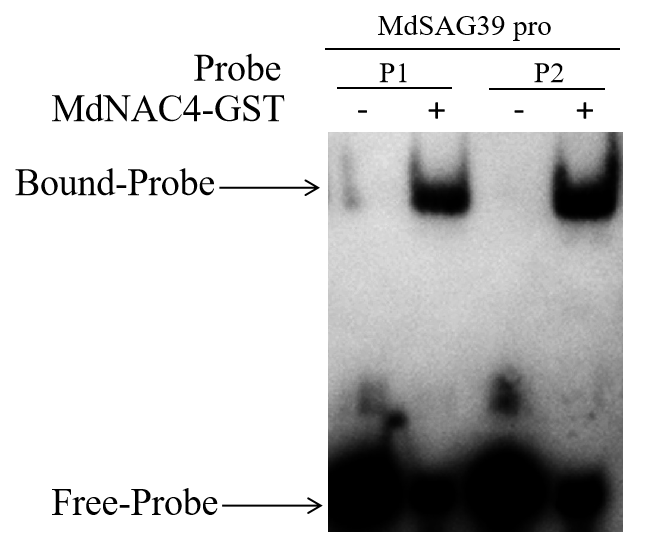


**Additional file 4: Fig. S4.** MdNAC4 binds specific sequences of the *MdSAG39* promoter.

Supplement: Supplementary file 4 — Additional file 4: Fig. S4. MdNAC4 binds specific sequences of the MdSAG39 promoter. [file 43897_2023_53_MOESM4_ESM.docx]
