# Supplementary material for: The NAC transcription factor MdNAC4 positively regulates nitrogen deficiency-induced leaf senescence by enhancing ABA biosynthesis in apple
Source: Mol Hortic. 2023 Mar 10;3:5. doi: 10.1186/s43897-023-00053-4 (PMC10514974; doi:10.1186/s43897-023-00053-4)
Supplement: Supplementary file 3 — Additional file 3: Fig. S3. Identification of transgenic tobacco overexpressing MdNAC4. [file 43897_2023_53_MOESM3_ESM.docx]

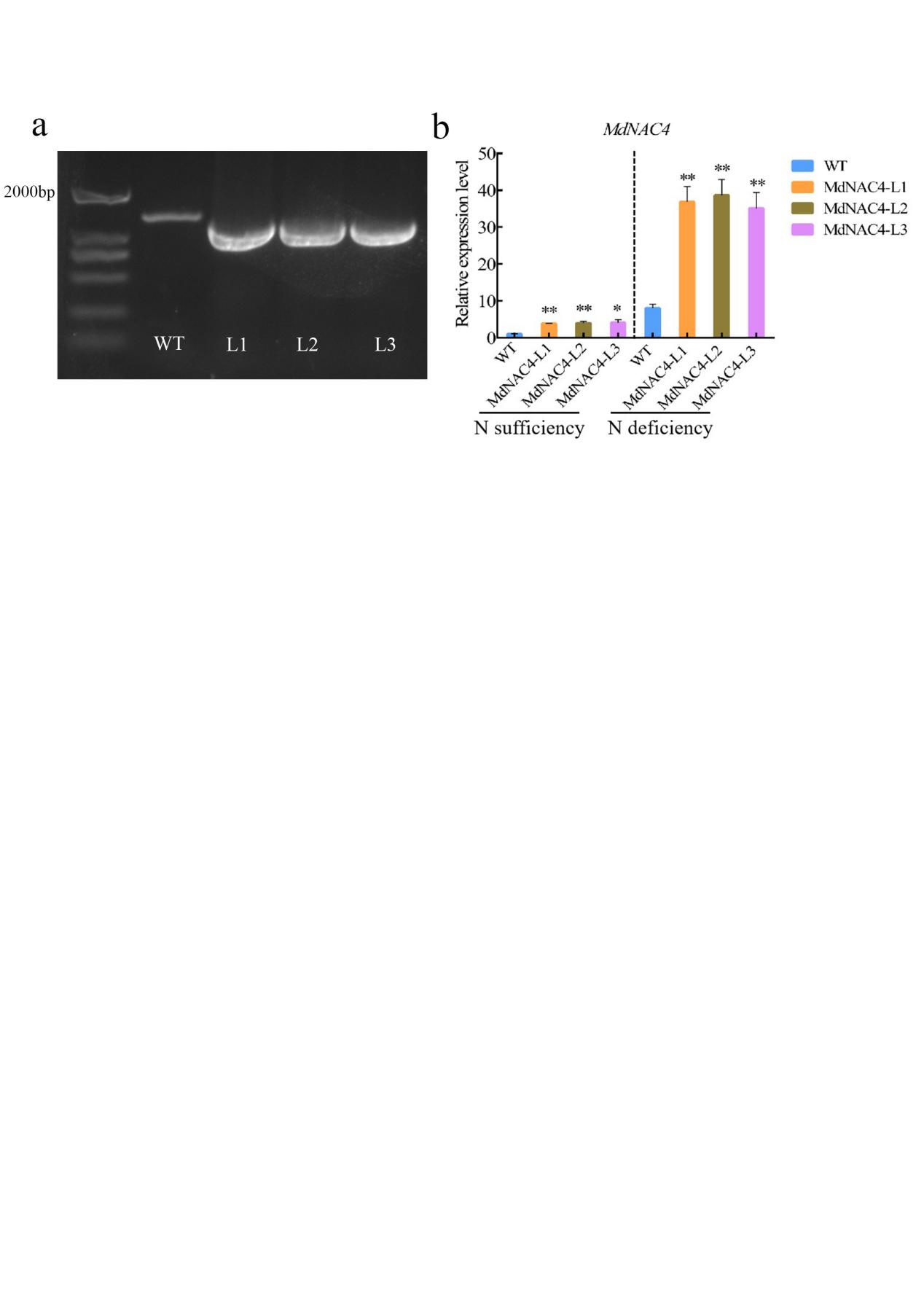


**Additional file 3: Fig. S3.** Identification of transgenic tobacco overexpressing *MdNAC4*.

(a) Identification by genomic PCR. (b) The expression of *MdNAC4* in wild-type (WT) and transgenic tobacco (MdNAC4-L1, MdNAC4-L2, MdNAC4-L3) under nitrogen-sufficient or nitrogen-deficient conditions. Under N-sufficient conditions, the N-sufficient WT was used as the control; under N-deficient conditions, the N-deficient wild type was used as the control. The expression level in the WT supplied with nitrate (+N) was set at 1. Error bars indicate the SDs of the three technical replicates and three biological replicates. Asterisks indicate significant differences between two independent samples according to t tests (*, P < 0.05 and **, P < 0.01).
