# Supplementary material for: The NAC transcription factor MdNAC4 positively regulates nitrogen deficiency-induced leaf senescence by enhancing ABA biosynthesis in apple
Source: Mol Hortic. 2023 Mar 10;3:5. doi: 10.1186/s43897-023-00053-4 (PMC10514974; doi:10.1186/s43897-023-00053-4)
Supplement: Supplementary file 5 — Additional file 5: Fig. S5. Expression level of MdNAC4 in WT and MdNAC4 transgenic tobacco plants after 30 μm ABA and -NO3− + 30 μm ABA treatment. [file 43897_2023_53_MOESM5_ESM.docx]

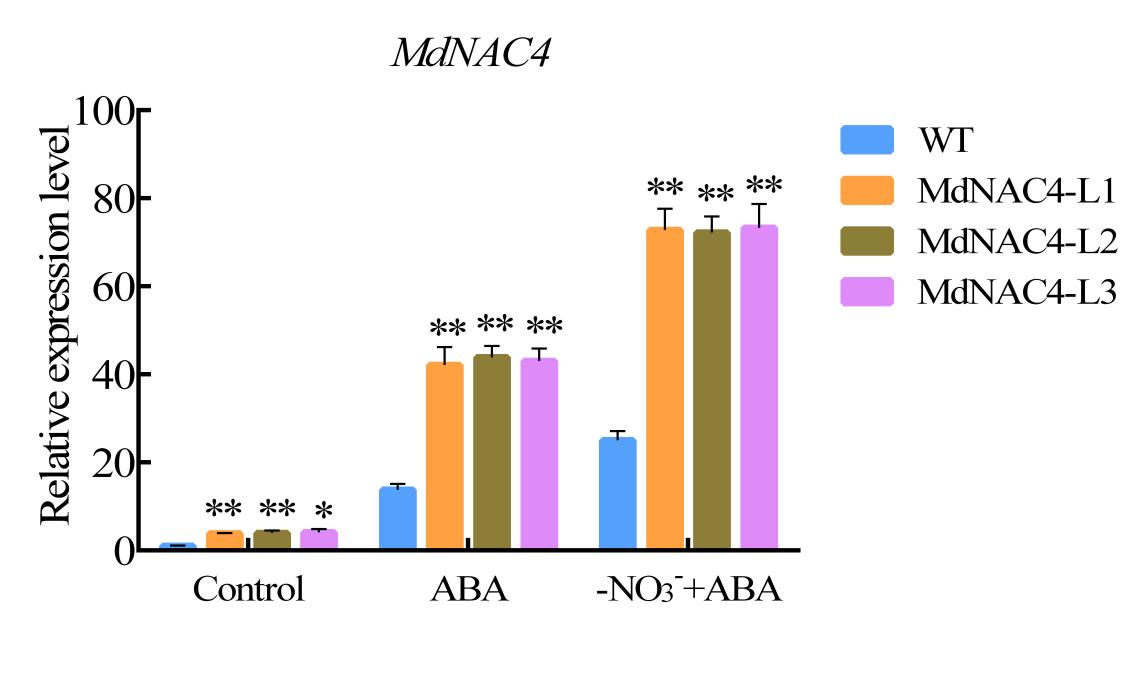


**Additional file 5: Fig. S5.** Expression level of *MdNAC4* in WT and *MdNAC4* transgenic tobacco plants after 30 μm ABA and -NO_3_^-^ + 30 μm ABA treatment. Untreated tobacco seedlings were used as controls. ABA, tobacco seedlings grown in Hoagland nutrient solution containing 30 μm ABA. -NO_3_^-^ + ABA, tobacco seedlings grown in nitrate-deficient Hoagland nutrient solution containing 30 μm ABA. The expression level in the untreated WT was set at 1. Error bars indicate the SDs of the three technical replicates and three biological replicates. Asterisks indicate significant differences between two independent samples according to t tests (*, P < 0.05 and **, P < 0.01).
