# Supplementary material for: The NAC transcription factor MdNAC4 positively regulates nitrogen deficiency-induced leaf senescence by enhancing ABA biosynthesis in apple
Source: Mol Hortic. 2023 Mar 10;3:5. doi: 10.1186/s43897-023-00053-4 (PMC10514974; doi:10.1186/s43897-023-00053-4)
Supplement: Supplementary file 6 — Additional file 6: Fig. S6. Identification of transgenic tobacco and apple seedlings overexpressing MdPYL4. [file 43897_2023_53_MOESM6_ESM.docx]

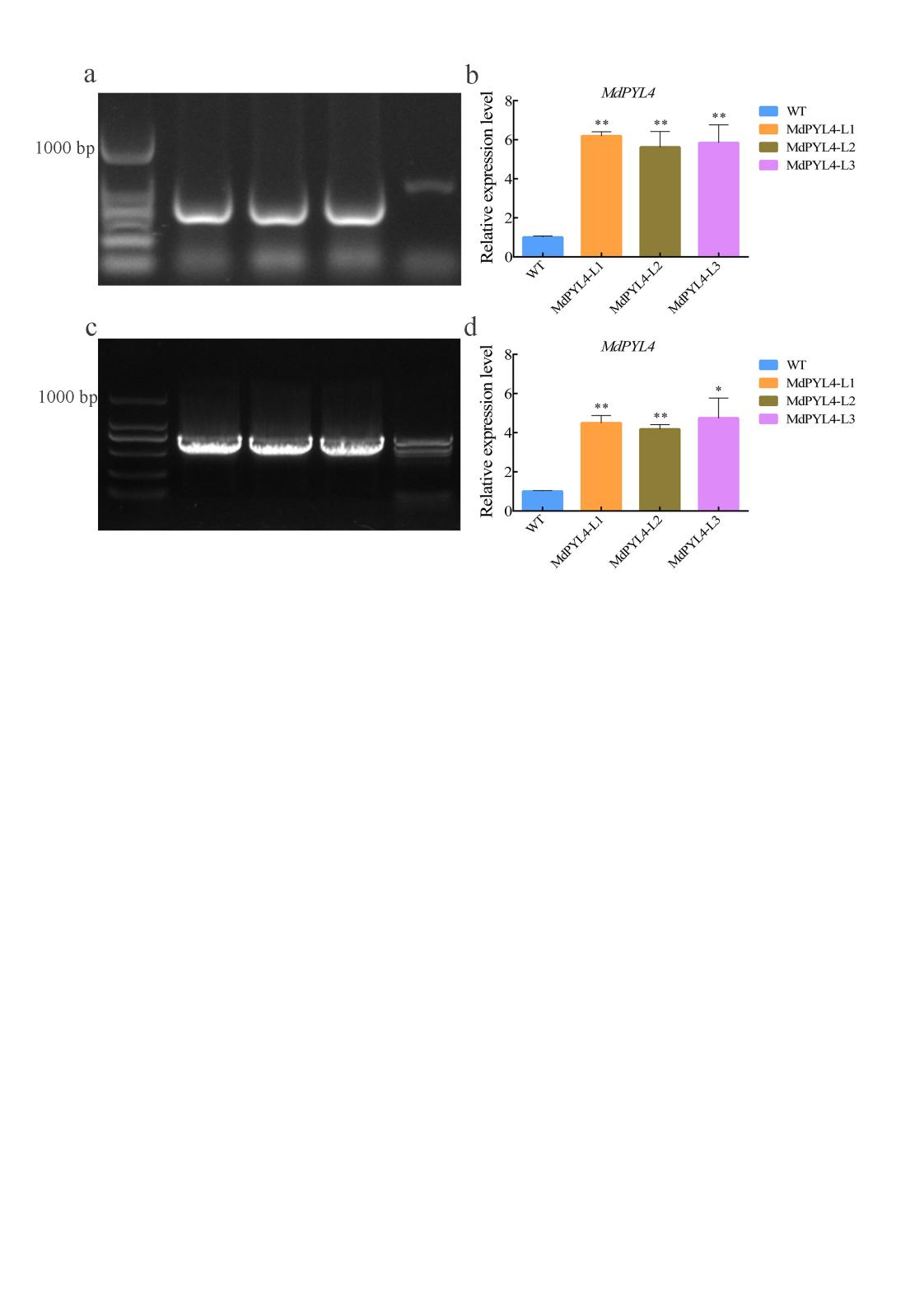


**Additional file 6: Fig. S6.** Identification of transgenic tobacco and apple seedlings overexpressing *MdPYL4*. Identification of transgenic tobacco (a) and apple seedlings (c) overexpressing *MdPYL4* by PCR. Identification of transgenic tobacco (b) and apple seedlings (d) overexpressing *MdPYL4* by qRT‒PCR. The WT expression level was set at 1. Error bars indicate the SDs of the three technical replicates and three biological replicates. Asterisks indicate significant differences between two independent samples according to t tests (*, P < 0.05 and **, P < 0.01).
