# Supplementary material for: The NAC transcription factor MdNAC4 positively regulates nitrogen deficiency-induced leaf senescence by enhancing ABA biosynthesis in apple
Source: Mol Hortic. 2023 Mar 10;3:5. doi: 10.1186/s43897-023-00053-4 (PMC10514974; doi:10.1186/s43897-023-00053-4)
Supplement: Supplementary file 7 — Additional file 7: Table S1. The primers used in this study. [file 43897_2023_53_MOESM7_ESM.docx]

**Additional file 7: Table S1** The primers used in this study

| **Name** | **Sequences (5’-3’)** | **Note** |
| --- | --- | --- |
| MdMAC4-F | ACTTGAGGCGTAGTGCGAAA | qRT-PCR prmers |
| MdNAC4-R | GTTCTATCGGAGCGGACGAG |  |
| MdGUSF | AGACTGTAACCACGCGTCTG |  |
| MdGUSR | TCCAGTTGCAACCACCTGTT |  |
| MdCYP707A1-F | GGTCACCAAGTTGAAGTGGGA |  |
| MdCYP707A1-R | TGAGAAATCAACTACATCGGCAG |  |
| MdZEP-F | ATCCTGCATAATCGGGAGCG |  |
| MdZEP-R | TGTAGCTGATACGAGCGTGC |  |
| MdNCED1-F | GGTGACGAGAAGTACGGTGG |  |
| MdNCED1-R | ATTTGCAGCTCCGACTTCCA |  |
| MdNCED2-F | ACGTTCTGCTTCCACCTCTG |  |
| MdNCED2-R | TTCTCGTCGCACTCGTTGAA |  |
| MdNCED6-F | AAAGGGGACGTGGACGAATC |  |
| MdNCED6-R | CACCTGCGTCCGAAATCTCT |  |
| MdNYC1-F | TCTTCGGTTTGGGGTGGTTT |  |
| MdNYC1-R | AACGCCTGTGCTCATCATGG |  |
| MdPAO-F | TCGCCCCCTTATCTGAAGGA |  |
| MdPAO-R | TCTTGGAGACCGAACAGCAC |  |
| MdSGR1-F | CCATATTTCCTGTTAATGCAAGGCT |  |
| MdSGR1-R | AGGTGCTTCTTTTCGTCCACT |  |
| MdSAG12-F | GGGCGAAGCTGGGTACATAA |  |
| MdSAG12-R | TGTGGGGTAAGAGGCAGACA |  |
| MdSAG29-F | ATCCGAACAAGAAGTGTCGAAT |  |
| MdSAG29-R | ATCTTCTATTATCACCTGCT |  |
| MdSAG39-F | GTGGTGTCTTCACCGGAACT |  |
| MdSAG39-R | TACCCAGTTTCGCCCCATTC |  |
| MdVNI2-F | GAGCAGCAAGTTCAACAGCC |  |
| MdVNI2-R | CTCGACCCTGAGGAGGAAGA |  |
| MdSINAT-F | TCCCTAGGAGTATCCGCGAC |  |
| MdSINAT-R | TGCCTATCCCCACCTGAGAA |  |
| MdBFN1-F | CGTTGGGGTGGGTGATACTC |  |
| MdBFN1-R | ACCGGAAGAAAAGACGCGAT |  |
| MdPYL4-F | AGGTATTCATCAATTCCCTCTTTGA |  |
| MdPYL4-R | AGGAGGAAAATTGGCCAAAATTAAA |  |
| NtSAG12-F | TGCAAGGCATGACCAATGGA |  |
| NtSAG12-R | ACCCTTTATCTTCACCTGCGT |  |
| NtSAG29-F | CACGGTACCAAATATTGCGGG |  |
| NtSAG29-R | GTTGTAGGCTGCTGCTCCTC |  |
| NtSAG39-F | TCATGGTGTTACAGCGGTTG |  |
| NtSAG39-R | TCCACAGAGTCCTTCCTCAGTA |  |
| OE-MdNAC4-F | ggactctagaggatccccgggATGGAAAATATTCTGGGTTTATTAACG | Full length primers of 35S::MdNAC4-GFP  Full length primers of 35S::MdPYL4-GFP |
| OE-MdNAC4-R | ataagggactgaccacccgggTCAATAATTCCAGAGGCAATCGA |  |
| OE-MdPYL4-F | ggactctagaggatccccgggATGTCTTCACCAATCCAGTTTCAA |  |
| OE-MdPYL4-R | gctcaccatggtacccccgggTCATGAGGGTTTCTTGGTGTTTG |  |
| IL60-MdNAC4-F | gcagaatctgaattcgtcgacATGGAAAATATTCTGGGTTTATTAACG | The overexpression pIR virus vector of 35S::MdNAC4 |
| IL60-MdNAC4-R | cccccacacgtgtggtctagaATAATTCCAGAGGCAATCGAATTC |  |
| TRV-MdNAC4-F | aaggttaccgaattctctagaGATACATATTTCAGGTTTGGTGAGG | The antisense TRV virus vector of 35S::MdNAC4 |
| TRV-MdNAC4-R | tgtcttcgggacatgcccgggAACAAGCGTAAGGAATGTCGT |  |
| BK-MdNAC4 (147-285 aa)-F | tcagaggaggacctgcatatgGCCTGTAATCTCCCCCAAA | Y2H primers |
| BK-MdNAC4 (147-285 aa)-R | tcgacggatccccgggaattcCAAAACTGAGTTTTGGTGCTGC |  |
| AD-MdNAC4-F | gtaccagattacgctcatatgATGGAAAATACTTCTGGGTT |  |
| AD-MdNAC4-R | atgcccacccgggtggaattcTCAATAATTCCAGAGGCAATC |  |
| AD-MdPYL4-F | gtaccagattacgctcatatgATGTCTTCACCAATCCAGTTTCAA |  |
| AD-MdPYL4-R | atgcccacccgggtggaattcTCATGAGGGTTTCTTGGTGTTTG |  |
| MdSAG39pro-F | agcacatgcctcgaggtcgacGATGTAATCACGAACGTTTGTA | The primers of MdSAG39 promoter |
| MdSAG39pro-R | agcacatgcctcgaggtcgacGGGTAGCTAGCTAGTGTAGTAGAAGC |  |
| GST-MdNAC4-F | gatctggttccgcgtggatccATGGAAAATATTCTGGGTTTATTAACG | Prokaryotic induction of proteins *in vitro* |
| GST-MdNAC4-R | gatgcggccgctcgagtcgacTCAATAATTCCAGAGGCAATCGA |  |
| HIS-MdNAC4-F | gccatggctgatatcggatccATGGAAAATATTCTGGGTTTATTAACG |  |
| HIS-MdNAC4-R | tgcggccgcaagcttgtcgacTCAATAATTCCAGAGGCAATCGA |  |
| GST-MdPYL4-F | gatctggttccgcgtggatccATGTCTTCACCAATCCAGTTTCAA |  |
| GST-MdPYL4-R | gatgcggccgctcgagtcgacTCATGAGGGTTTCTTGGTGTTTG |  |
| MdNAC4^NC^-F | agaacacggggggactctagaATGGAAAATACTTCTGGGT | BiFC primers |
| MdNAC4^NC^-R | gacagtactatcgatggatccATAATTCCAGAGGCAATCGA |  |
| MdPYL4^NC^-F | agaacacggggggactctagaATGTCTTCACCAATCCAGTTTC |  |
| MdPYL4^NC^-R | gacagtactatcgatggatccTCATGAGGGTTTCTTGGTGTTT |  |
| 62SK-MdNAC4-F | cgctctagaactagtggatccATGGAAAATATTCTGGGTTTATTAACG | Dual luciferase primers |
| 62SK-MdNAC4-R | gggccccccctcgaggtcgacTCAATAATTCCAGAGGCAATCGA |  |
| LUC-MdNCED2-F | gggccccccctcgaggtcgacGTGGGACCGTGGGAGGGA |  |
| LUC-MdNCED2-R | cgctctagaactagtggatccGAAGTTGTTTATTTGTGAAGGAGGTC |  |
| LUC-SAG39-F | gggccccccctcgaggtcgacTAAAAAATTATTAAAAAGATATA |  |
| LUC-SAG39-R | cgctctagaactagtggatccCGCGTCCGCGTGAGTATG |  |
| p-MdNCED2-F | TAAATCCAC**CACGTG**GTCTCTCATG | Probe for EMSA primers |
| p-MdNCED2-R | CATGAGAGAC**CACGTG**GTGG ATTTA |  |
| p-MdSAG39-F | TTTTGTAAGG**CACGT**ACCACGCTGA |  |
| p-MdSAG39-R | TCAGCGTGGT**ACGTG**CCTTA CAAAA |  |
| Mut-MdNCED2-F | TAAATCCAC**GACGGG**GTCTCTCATG |  |
| Mut-MdNCED2-R | CATGAGAGAC**CCCGTC**GTGG ATTTA |  |
| Mut-MdSAG39-F | TTTTGTAAGG**GACGG**ACCACGCTGA |  |
| Mut-MdSAG39-R | TCAGCGTGGT**CCGTC**CCTTA CAAAA |  |
